# Impact of food-based fortification on nutritional outcomes and acceptability in older adults: systematic literature review

**DOI:** 10.3389/fnut.2023.1232502

**Published:** 2023-10-27

**Authors:** Alexia Geny, Maité Petitjean, Virginie Van Wymelbeke-Delannoy, Claire Sulmont-Rossé

**Affiliations:** ^1^Centre des Sciences du Goût et de l'Alimentation, CNRS, INRAE, Institut Agro, Université de Bourgogne, Dijon, France; ^2^CHU Dijon Bourgogne, Unité de recherche Pôle Personnes Âgées, Dijon, France

**Keywords:** elderly, enrichment, supplementation, food-first, malnutrition, intake, body weight, acceptability

## Abstract

**Background:**

“Do it yourself” (DIY) food-based fortification involves adding fortificants into everyday foods. It is a flexible solution that allows older people with reduced appetite to meet their nutritional needs.

**Objectives:**

The aims of the systematic review are (a) to describe DIY fortified recipes, (b) to evaluate their acceptability, and (c) to evaluate whether they are effective levers to improve nutritional outcomes in older people.

**Methods:**

A systematic search of 3 databases (Web of Science, PubMed, Scopus, last searched on January 2022) was undertaken. Main eligibility criteria include older adults aged ≥60 years living at home, in an institution or in hospital. Studies carried out for a specific medical condition or targeting only micronutrient fortification were excluded. After reviewing all titles/abstracts then full-text papers, key data were extracted and synthesized narratively. The quality of included studies was assessed using Kmet et al.

**Results:**

Of 21,493 papers extracted, 44 original studies were included (3,384 participants), with 31 reporting nutritional outcomes, 3 reporting acceptability outcomes and 10 reporting both nutritional and acceptability outcomes. The review highlighted a wide variety of DIY fortified recipes, with additional energy ranging from 23 to 850 kcal/d (*M* = 403; SE = 62) and/or protein ranging from 4 to 40 g/d (*M* = 19; SE = 2). Compared to a standard diet, DIY fortification seems to be a valuable strategy for increasing energy and protein intake in older people. However, no strong evidence was observed on the nutritional status.

**Implication for future:**

Further acceptability studies are crucial to ensure that DIY fortified foods are palatable and thus have a significant impact on the nutritional status. In addition, it would be useful for studies to better describe DIY recipes. This information would result in a better understanding of the factors that maximize the impact of DIY fortification on nutritional outcomes. Study registration: PROSPERO no. CRD42021244689.

**Systematic review registration**: PROSPERO: https://www.crd.york.ac.uk/prospero/display_record.php?ID=CRD42021244689.

## 1. Introduction

Contrary to common beliefs, our nutritional needs decrease little with age and are sometimes higher in late adulthood than in early adulthood. With regard to caloric intake, the European Food Safety Authority (1) recommends a daily allowance from 2000 to 2,500 Kcal for people aged 50 to 59 and from 1800 to 2,300 Kcal for people aged 70 to 79. More recently Volkert et al. (2) established that recommended energy intake should reach 30 Kcal per kg of body weight per day. With regard to protein intake, recent works carried out by the PROT-AGE consortium (3) and by the European Society for Clinical Nutrition and Metabolism (EPSEN) (4) show that older people need to ingest more protein than younger people to stay healthy, to maintain their abilities and to fight infections. As a result, the daily protein intake should be 1 to 1.2 g protein per kg of body weight per day for a healthy person over 60 versus 0.8 to 1 g per kg of body weight in younger adults. The literature review by Shad et al. (5) highlighted the importance of a constant distribution of protein intake over the main meals of the day at amounts of 25–30 g/meal to avoid catabolic protein status [see also (3, 6)].

At the same time, a decline in appetite can appear with aging (7). Various studies have reported that 31 to 56% of the aged population are “small eaters” (8–10). Small eaters are characterized by a low consumption of every food category compared to the overall population – they eat foods in small or even very small amounts (8–11). A recent French survey carried out by CREDOC (“Centre de Recherche pour l’Observation et les Conditions de Vie”) showed that 87% of adults aged 18–54 met the recommendations for protein intake compared with only 56% of those over 65 (12). This situation is even worse when older adults are frail and dependent. In an aged population receiving a Home-Delivery Meal (HDM) service or living in nursing homes, Sulmont-Rossé and Van Wymelbeke (13) observed that 7–8 out of 10 people did not meet their energy and/or protein needs. This study also showed that 55% of home-delivery meal recipients and 46% of people living in nursing homes had energy and/or protein intake lower than 2/3 of the recommendations. In addition to age, many factors can be at the origin of this decline in appetite, such as physiological changes, sensory decline and eating/swallowing difficulties, which appear during aging. It also can be related to “life-breaking moments” (e.g., widowhood, illness, dependence) that can amplify iatrogenic factors correlated with medications and affect sociological/psychological aspects (13). Thus, poor appetite in older adults leads to a decrease in food and nutrient intake, which increases the risk of undernutrition (14, 15). Undernutrition, a recognized pathology in the older population, corresponds to an imbalance between nutritional intake and the body’s needs. This imbalance leads to weight loss, a decrease in muscle reserves and an alteration of the body’s defences. In older people, undernutrition increases the risk of falls and therefore fractures. It contributes to the increase in infectious morbidity (16), nosocomial infections (17) and the appearance of pressure ulcers (18). If left untreated, undernutrition can induce or aggravate a state of fragility and dependence, which affects the quality of life and life expectancy of our elders (16, 19).

Understanding the factors responsible for appetite decline is certainly important, but a major challenge is to get older people with reduced appetite to fulfill their nutritional needs in order to prevent undernutrition and the associated consequences. Food-based fortification, which consists in incorporating ingredients of nutritional interest (namely “fortificants”) in commonly consumed foods (20) in order to deliberately increasing the content of an essential nutrient in a diet without increasing (too much) the volume to be ingested, is acknowledged to be a relevant approach for older adults with reduced appetite (21). Fortificants can be: (a) regular food products (e.g., semolina, oils, butter, cream, pureed nuts, egg), or (b) macronutrients extracts (e.g., whey protein isolate, milk protein concentrate, caseinate, maltodextrin) (22, 23). Besides the numerous fortified foods developed and marketed by the food industry, “do it yourself” (DIY) fortification recipes empower older adults and their carers to take a personalized approach to their nutrition and current diet. DIY fortification is a flexible strategy that may fit better with older people’s food habits and preferences: older people (or their carers) add fortificants to the food they usually eat, during the preparation of daily meals. This constitutes a significant advantage in the older population, which is often reluctant to change their consumption habits. However, DIY fortification remains largely unknown and underused by older adults as well as by caregivers and healthcare professionals although it is now known to be a relevant approach to counterbalance appetite decline and to adjust to nutritional needs (24).

The goal of the present study was to conduct a systematic review of all studies related to the nutritional and acceptability aspects of DIY food-based fortification in older people. The aims of this review are (a) to describe the DIY food-based fortification solutions and recipes that have been developed, (b) to evaluate the acceptability of these solutions in older people, and (c) to evaluate whether these solutions can be relevant and effective levers to preserve or improve nutritional outcomes in older people.

## 2. Materials and methods

The present systematic review followed the approach proposed by Xiao and Watson (25), which summarizes the evidence available on a topic to convey the breadth and depth of that topic. The protocol was written using the Preferred Reporting Items for Systematic Reviews and Meta-analysis Protocols (PRISMA-P, (26), see Supplementary material). The protocol was deposited on the HAL website^1^ and on PROSPERO with the registration number CRD42021244689. The PRISMA checklist is available on the Supplementary material.

### 2.1. Research question

The research question is: “What are the objectives, characteristics and results of existing research conducted on the nutritional issues and/or on acceptability among older people receiving DIY fortified foods?”

### 2.2. Inclusion and exclusion criteria

The PICOS (Population, Intervention, Comparator, Outcome, Study design) eligibility criteria was as follows (27):

*Population:* Any studies focusing on adults aged 60 years and older living either at home, in an institution or in hospital was eligible for inclusion. Older adults of all nutritional status, cognitive status and oral ability (e.g., chewing, swallowing) were eligible for inclusion. Studies carried out in the context of a specific medical condition (e.g., cardiac rehabilitation, renal failure, cancers, diabetes) were excluded.

*Intervention:* Any DIY food-based fortification intervention was eligible for inclusion (e.g., incorporating ingredients of nutritional interest in food products). Fortification in energy and/or macronutrients was eligible for inclusion. Studies without an intervention (e.g., observational studies) were relevant for inclusion. Were excluded from the review: (a) studies targeting only micronutrient fortification, non-food dietary supplement or bio-fortification (genetically modified crop), (b) studies using only fortified food developed and marketed by the Food Industry, and (c) interventions targeting artificial nutrition (e.g., tube feeding, parenteral feeding, enteral feeding).

*Comparators:* As the present review aimed at compiling DIY food-based fortification recipes and reporting their acceptability, any comparator was eligible for inclusion (e.g., studies comparing food-based fortification versus Oral Nutritional Supplements (ONS), or studies comparing two types of fortified food). In addition, studies without a comparator were eligible for inclusion.

*Outcomes:* Three categories of outcomes were considered: (a) characterization of the nutritional intake (e.g., dietary pattern, nutrient intake), (b) characterization of the nutritional status (e.g., body mass index (BMI), weight, undernutrition) and (c) characterization of the acceptability (e.g., liking, preference, pleasure).

*Study design:* All types of study design including interventional and observational design were eligible. All period of times and duration of follow-up were eligible.

*Other:* No restriction was set for the publication date. Only publications written in English were included because of the uncertainty surrounding the words used to refer to the concept of “DIY food-based fortification” in foreign languages. Narrative review, conference abstracts, editorials, and grey literature were excluded.

### 2.3. Information sources and search strategy

A search strategy with both thesaurus and free-text terms was developed – after repeated attempts and adjustments – to retrieve relevant articles in the following databases: Web of Science (WOS), PubMed and Scopus (Supplementary material). Separate title, abstract and keywords searches were conducted for older people, food-based fortification and outcomes in February 2021. An update was performed in January 2022. The results for the three separate search strings were combined to identify relevant articles. Afterwards, for further screening, references from selected articles and systematic reviews were checked manually in case they were not identified during the whole search process. After duplicates removal, titles and abstracts in the first step and full texts in a second step were screened by two independent reviewers (AG and MP) according to the agreed inclusion and exclusion criteria. For each screening level, a training exercise was conducted before the starting of the screening process on a random sample of 100 titles and abstracts and 10 full texts to ensure high inter-reviewer reliability. Disagreements between reviewers were resolved by consensus or by consulting a third reviewer (CSR or VVW). The reasons for exclusion were recorded at the full-text stage (the list of excluded studies at the full-text stage and the reasons of exclusion are presented on Supplementary material).

### 2.4. Charting the data

A standardized data summarization form was developed *a priori* and revised, as needed, after the completion of a training exercise completed on a sample of 5 articles. All included studies were summarized by two reviewers (AG and MP), independently, with conflicts resolved by a third reviewer (CSR or VVW). The data summarization included the following items:

- Article identifiers (authors, year of publication)
- Study identifiers (objective, design, country)
- Population (age, gender, sample size, inclusion and exclusion criteria)
- Intervention (description of the DIY fortification recipes)
- Comparator (if applicable)
- Outcomes (endpoints, measurement method, main results)

### 2.5. Quality assessments

All included studies were independently assessed for quality by two reviewers (AG and MP); conflicts were resolved by consensus. The articles’ quality was assessed with the quality assessment criteria developed by Kmet et al. (28). The criteria are presented in Supplementary material. In addition, the description quality of the DIY fortification recipes (fortificants, food matrices, concentration) was assessed (but not included in the quality score).

### 2.6. Collating, summarizing and reporting the results

A descriptive numerical summary of the characteristics of the included studies was performed. Tables and graphs were created to reflect the number of studies included, study designs and settings, publication years, the characteristics of the study populations, the outcomes reported, and the countries where the studies were conducted. In line with systematic literature review guidelines, the quality of the included studies was assessed (25, 29).

## 3. Results

### 3.1. Characteristics of the included studies

**Figure 1:**
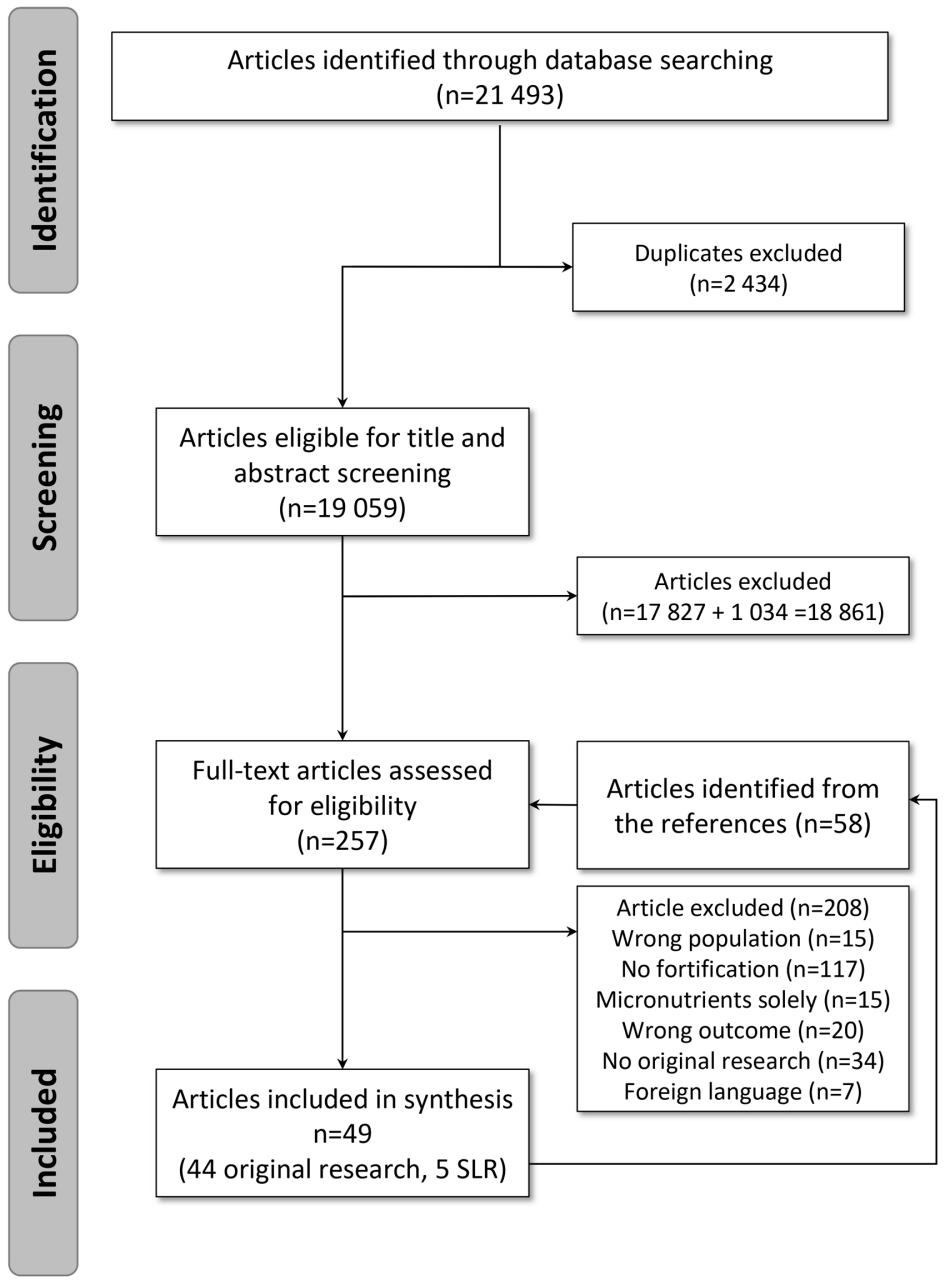
PRISMA flow diagram.

On the 21,493 articles retrieved, 253 records were kept for full text screening and 49 studies were included in the systematic review: 44 original studies (Figure 1; 3,384 participants) and 5 systematic literature reviews (21–24, 30). The reasons for excluding papers were: no original research (*n* = 18), wrong population (*n* = 18), no DIY food-based fortification (*n* = 135), fortification with micronutrients only (*n* = 15), wrong outcomes (*n* = 18). Wrong outcomes included functional outcomes (muscle strength), gastric emptying, glycemia, gut hormones, bone mineral density, quality of life. Two articles (31, 32) were excluded because they did not provide enough information about the nutritional strategy used.

The included articles were published between 1996 and 2021, and most were published after 2011 (*n* = 34) (Table 1). The studies mainly took place in Europe (*n* = 33). The rest took place in Australia (*n* = 4), North America (*n* = 4) or Asia (*n* = 3). The setting was most often the hospital (*n* = 20) followed by nursing homes (*n* = 13) and home setting (*n* = 13). Twenty-seven studies of the selection were longitudinal with follow-up times between 10 days to 12 months and 16 studies were cross-sectional (Table 1). In addition, 30 studies used a between-subject design while 13 studies used a within-subject design; only 1 study was observational. Finally, sample sizes varied (ranging from 7 to 320 participants), but most studies recruited 20 to 49 subjects (*n* = 17).

**Table 1:** Characteristics of the systematic literature review articles.

	Nb	%
Year of publication		
1990–2000	3	6.8%
2001–2010	7	15.9%
2011–2021	34	77.3%
Design		
Longitudinal study	27	62.8%
Of which < 1 month	7	16.3%
Of which 1–3 months	15	34.9%
Of which > 3 months	5	11.6%
Cross-sectional survey	16	37.2%
Between-subject	30	68.2%
Within-subject	13	29.5%
Observational	1	2.3%
Country		
Australia	4	9.1%
Belgium	1	2.3%
Canada	2	4.5%
Denmark	5	11.4%
Finland	3	6.8%
France	2	4.5%
Germany	3	6.8%
Japan	1	2.3%
Korea	1	2.3%
Netherlands	8	18.2%
Sweden	4	9.1%
Switzerland	1	2.3%
Taiwan	1	2.3%
UK	6	13.6%
USA	2	4.5%
Setting		
Home	13	28.3%
Of which home care	2	4.3%
Of which HDM	2	4.3%
Hospital	20	43.5%
Nursing home	13	28.3%
Nb participants		
>200	2	4.4%
100–200	8	17.8%
50–99	14	31.1%
20–49	17	37.8%
<20	4	8.9%

HDM, home-delivery meal.

Among the 44 original research studies, 3 were fully focused on the acceptability outcome (33–35). Among the 41 remaining articles, the majority (*n* = 31) were entirely dedicated to nutritional outcomes. Finally, 10 articles were “mixed” and assessed both nutritional and acceptability outcomes.

A descriptive summary of the included studies yielded the four following topics:

- Description of DIY fortification recipes: which types of food are fortified? Which nutrients are added? In what form? At which concentration?
- Assessment of DIY fortified foods acceptability: to which extent do older people like fortified food? Do the sensory characteristics of fortified foods fulfil older people’ sensory expectations and preferences?
- Assessment of the nutritional impact of DIY food-based fortification: did older people who received fortified food improve their nutritional intake and nutritional status compared to a standard diet?
- Comparison of DIY food-based fortification with other alternatives (e.g., dietary counseling, Oral Nutritional Supplement – ONS): is fortified food more acceptable and/or does it provide a nutritional benefit compared to other alternatives?

### 3.2. Quality assessment

A quality assessment was performed for each outcome, i.e., nutritional outcome and acceptability outcome (Supplementary material). In fact, in mixed articles, different panels and designs were often used for nutritional and acceptability outcomes.

Regarding nutritional outcomes, the methodological quality of the studies was in general good with an average quality score of 0.92 (standard deviation: 0.09) ranging from 0.62 (36) to 1 (37–54) (Supplementary material). Overall, recruitment of participants was the variable that was the most poorly rated in the selected studies. This was because the majority of studies did not detail the recruitment procedure nor the precise localization where the study took place. Sample size and control for confounding factors were badly rated because a large number of studies did not reach an appropriate sample size or did not consider confounding variables (e.g., age, gender, Body Mass Index (BMI), weight, nutrition status) in data analysis. Study design and subject description factors were moderately rated due to insufficient/incoherent information preventing clear understanding of concerned articles.

The methodological quality of the 13 studies related to acceptability outcomes was on the whole lower than for the nutritional outcomes, with an average quality score of 0.75 (standard deviation: 0.23) ranging from 0.33 (37) to 1 (33, 43, 44) (Supplementary material). Usually, recruitment of participants, sample size, analytic methods and results were the lowest rated factors. As for the nutritional quality assessment, the majority of studies did not detail the recruitment procedure nor the precise localization where the study took place. Moreover, most studies did not clearly describe the analytic method used when it was mentioned. For 4 criteria (sample size, results, outcomes measures and study design) the poor quality is related to the fact that the acceptability measure was not the main outcome of the article.

Finally, the description of the DIY fortification recipes was also poorly rated: very few studies provided precise information about food matrices, fortificants and recipes.

### 3.3. Description of DIY fortified recipes

Table 2 shows the description of the DIY fortified recipes. On the whole, 7 articles implemented energy fortification, 18 implemented protein fortification and 19 implemented a combination of protein and energy fortification. It should be noted that 10 articles did not specify the nature of food matrices (38, 44, 55, 58, 61, 64, 65, 67, 72, 74) and 5 articles did not specify the nature of the fortificants (50, 53, 56, 57, 74). Only 8 articles provided enough details about the recipes for them to be reproduced by a third party (33–35, 39, 46, 49, 54, 73).

**Table 2:** Description of DIY fortified recipes.

Author(s) (year), Country	Population	Type of fortification	Matrices	Fortificants	Target meal	Additional supply from fortification	Detailed recipe given?
Allepaerts et al. (2020) (53), Belgium	Hospital (*n* = 78) 85 y 77% of women	Energy	Homemade cream snack, soup	Not specified	Snack, lunch and dinner	+ 540 kcal/d + 24 g proteins/d	No
Arjuna et al. (2018) (48), Australia	Home with HDM (*n* = 29) 83 y 55% of women	Protein & Energy	Soup, dessert Sauces	+ Skim-milk protein or cream or custard + Extra cheese or margarine or oil	Lunch	+ 550 kcal/d + 30 g proteins/d	No
Barton et al. (2000) (55), UK	Hospital (*n* = 35) 77 y 63% of women	Energy	Meals	+ Fats (butter, cream and cheese) + Carbohydrates (glucose polymers)	Day	+ 200 kcal/d - 5 g proteins/d - 20% portion size/meal	No
Beelen et al. (2017a) (45), Netherlands	Hospital and nursing home (*n* = 22) 83 y 59% of women	Protein	Bread, soup, fruit juice, mashed potatoes	+ Soy or dairy proteins	Day	Not specified	No
Beelen et al. (2017b) (56), Netherlands	Home (*n* = 75) 77 y 56% of women	Protein	Bread, meatballs, dairy dessert	Not specified	Day	Not specified	No
Beelen et al. (2018) (57), Netherlands	Hospital (*n* = 147) 79 y 55% of women	Protein	Bread, soup, beverages, beef, mashed potatoes, ice cream	Not specified	Day	Not specified	No
Beermann et al. (2016) (42), Denmark	Hospital (*n* = 62) 69 y Not specified	Protein	Skyr Yoghurt, oatmeal Omelet	+ Cream + WPI + Cheese, ham	Breakfast	Maximum intake: 20 g proteins/breakfast	No
Björkman et al. (2012) (39), Finland	Nursing home (*n* = 99) 84 y 76% of women	Protein	Fruit juice	+ Whey protein	Day	+ 20 g proteins/d	Yes
Bonnefoy et al. (2010) (58), France	Hospital (*n* = 26) 81 y 58% of women	Protein	Liquid food, semi-liquid food	+ Hyperprotidine powder (BCAAs)	Lunch and dinner	+ 11–18 g proteins/d (of which 47.5% BCAAs)	No
Castellanos et al. (2009) (59), USA	Nursing home (*n* = 26) 87 y 70% of women	Protein & Energy	Oatmeal Soup Potato side dish	+ Fats + Sugar + Fats + Starchy ingredients + Fats Fats (margarine, high-fat dairy products and kosher non-dairy substitute…) Proteins (dairy or eggs)	Breakfast and lunch	+ 4.17 kcal/g food + 0.06 g protein/g food	No
Evans et al. (2017) (46), Canada	Home (*n* = 41) 60 y 64% of women	Protein	Orange juice	+ L-carnitine combination sachet or + L-carnitine sachet	Breakfast	+ 1.5–6.5 g proteins/d	Yes
Gall et al. (1998) (60), UK	Hospital (*n* = 143) 67 y 66% of women	Protein & Energy	Dessert Soup	+ Double cream + Dried skimmed-milk or milk powder	Lunch and dinner	Not specified	No
Hashimoto et al. (2015) (61), Japan	Hospital (*n* = 28) 74 y 57% of women	Protein	Meals	+ Casein powder or + Soy protein isolate	Lunch	+ 7.1–7.5 g proteins/d	No
Irvine et al. (2004) (36), France	Hospital (*n* = 12) 84 y 33% of women	Protein & Energy	Semi-skimmed milk	+ Fresh cream, sugar, dextrin maltose or + Protifar protein powder, sugar, dextrin maltose	Breakfast	+ 250 kcal/d + 3.5–20 g proteins/d	No
Iuliano et al. (2013) (62), Australia	Nursing home (*n* = 130) 88 y 78% of women	Protein & Energy	Soup Vegetables	+ Milk powder or evaporated milk or cheese + Cheese-based sauces	Day	Not specified	No
Lee et al. (2013) (40), Taiwan	Nursing home (*n* = 83) 80 y 58% of women	Protein	Warm drink	+ Soy powder	Snack	+ 250 kcal/d + 9.5 g proteins/d	No
Leslie et al. (2013) (63), UK	Nursing home (*n* = 31) 91 y 88% of women	Energy	Cereal, porridge, soup, dessert Potatoes Malted milk snack	+ Double cream + Butter Replace water by whole milk	Day	+ 400 kcal/d	No
Lorefält et al. (2005) (64), Sweden	Hospital (*n* = 10) 82 y 60% of women	Protein & Energy	Meals	+ Fats: cream, butter, mono and poly unsaturated oils + Proteins: gruels of maize	Lunch and dinner	+ 0 kcal/d + 0 g proteins/d – 50% portion size	No
Mertz et al. (2021) (65), Denmark	Home (*n* = 184) 70 y 46% of women	Protein	Fluids	+ Protein powder (whey or collagen)	Breakfast and lunch	+ 40 g protein/d	No
Mortensen et al. (2019) (51), Denmark	Hospital (*n* = 92) 69 y 56% of women	Protein	Milkshake, chocolate cake, pizza bun, fruit salad, bun, cheese crackers, sandwich, jelly	+ Egg or shun + Whey protein or gelatine or pea protein	Snack	18–27 kcal/d 15–23 g proteins/d	No
Munk et al. (2013) (66), Denmark	Hospital (*n* = 79) 73 y 75% of women	Energy	Meat, fish, egg, vegetables, soup, cereal, pulse, bread, dairy, beverage, dessert	+ Fats (butter, cream, oil…)	Day	Not specified	No
Munk et al. (2014) (41), Denmark	Hospital (*n* = 78) 75 y 58% of women	Protein & Energy	Meat, fish, egg, vegetables, soup, cereal, dessert	+ Natural energy-dense ingredients + High-quality protein powder GlanPro	Day	+ 0.6–4.7 kcal/g food + 6.1–11.5 g proteins/serving	No
Neelemaat et al. (2012) (67), Netherlands	Hospital + Home (*n* = 150) 75 y 55% of women	Protein & Energy	Oatmeal, desserts Dishes	+ Cream, maltodextrin + Milk products or butter or margarine	Day	+ 750 kcal/d + 30 g proteins/d	No
Niccoli et al. (2017) (47), Canada	Hospital (*n* = 47) 81 y 68% of women	Protein	Porridge, milk based-drink	+ Whey protein	Day	+ 24 g proteins/d	No
Norton et al. (2020) (33), UK	Home (*n* = 32) 75 y 56% of women	Protein	Cupcakes	+ WPC or WPe	Snack	+ 6 g proteins/100 g	Yes
	Home (*n* = 42) 74 y 55% of women	Protein	Cake, biscuits	+ WPI	Snack	Cake: + 6 g proteins/100 g Biscuit: + 10 g proteins/100 g	Yes
Nykänen et al. (2019) (52), Finland	Home with home care (*n* = 85) 83 y 72% of women	Energy	Berry purée	+ Sugar, rapeseed oil	Snack	Not specified	No
Ödlund Olin (2003) (69), Sweden	Nursing home (*n* = 35) 82 y 51% of women	Energy	Beef in horseradish sauce Fruit syrup dessert Oven-baked sausage Mashed potatoes, boiled broccoli	Whipping cream instead of milk + Hydrolysed starch, cream instead of milk + Cheese + Margarine	Lunch and dinner	+ 500 kcal/d	No
Ödlund Olin et al. (1996) (70), Sweden	Hospital (*n* = 36) 82 y 67% of women	Energy	Vegetable casserole Rosehip soup Fish quenelle Fish soup, pancake with jam Ragout with liver Potatoes Beans	+ Oil, cream + Almond biscuit + Sour cream + Cream + Sour cream + Milk + Margarine	Lunch and dinner	+ 850 kcal/d	No
Ott et al. (2019) (71), Germany	Nursing home (*n* = 16) 87 y 88% of women	Protein & Energy	Cream, mousse Vegetable Meat, fish, smoothie	+ Whey protein + Rapeseed oil + Rapeseed oil, whey protein	Day	+ 600 kcal/d + 30 g proteins/d	No
Park et al. (2018) (49), Korea	Home (*n* = 99) 77 y 65% of women	Protein	Corn silk tea	+ Whey protein	Snack	+ 0.4–0.7 g proteins/kg/d	Yes
Polonen et al. (2017) (72), Finland	Home with home care (*n* = 227) 84 y 71% of women	Protein & Energy	Food Bread	+ Oils + Margarine or cheese	Day	Not specified	No
Seemer et al. (2021) (54), Germany	Nursing home (*n* = 50) 84 y 74% of women	Protein	Cream (sweet or savory version)	+ Whey protein	Lunch	+ 125–250 kcal/d + 10–20 g protein/d	Yes
Silver et al. (2008) (37), USA	Home with HDM (*n* = 45) 84 y 69% of women	Protein & Energy	Mashed potatoes Broccoli casserole	+ Eggs and replacing water by non-dairy kosher creamer + Almonds, mayonnaise	Lunch	+ 300 kcal/d + 10 g proteins/serving	No
Smoliner et al. (2008) (73), Germany	Nursing home (*n* = 52) 83 y 73% of women	Protein & Energy	Soups Sauces Milk basis snack	+ Hydrolyzed milk, heavy cream + Hydrolyzed milk, rapeseed oil + Hydrolyzed milk	Day	Not specified	Yes
Sossen et al. (2020) (74), Australia	Nursing home (*n* = 122) 88 y 76% of women	Protein & Energy	Milkshake, fruit juice, milk, porridge Meals	Not specified + Butter	Day	+ 701 kcal/d + 27 g proteins/d	No
Starke et al. (2011) (38), Switzerland	Hospital (*n* = 132) 73 y Not specified	Protein & Energy	Meal	+ Maltodextrin + Rapeseed oil + Cream and/or protein powder	Day	Not specified	No
Stelten et al. (2015) (75), Netherlands	Hospital (*n* = 47) 80 y 55% of women	Protein	Drinking yoghurt	+ WPC	Day	+ 13 g proteins/serving (*ad libitum*)	No
Stow et al. (2015) (76), UK	Nursing home (*n* = 67) Not specified 82% of women	Protein & Energy	Fruit, dessert, dairy, beverage	+ Milk powder, cream	Day	+ 600 kcal/d + 20–25 g proteins/d	No
Tsikritzi et al. (2015) (34), UK	Home (*n* = 67) 71 y Not specified	Protein & Energy	Sauces	+ Unsalted butter or + Double cream or + WPI, maltodextrin or + Whole milk, double cream or + Double cream, vegetable oil, unsalted butter	Sauce	+ 69–150 kcal/100 g + 0.0–1.3 g proteins/100 g	Yes
Van Til et al. (2015) (77), Netherlands	Hospital (*n* = 34) 78 y 68% of women	Protein	Drinking yoghurt	+ WPC	Day	+ 25 g proteins/d	No
Wendin et al. (2017) (35), Sweden	Home (*n* = 7) 60–69 y 71% of women	Protein	Muffin	+ Almond flour or + Soy flour or + Whey protein	Snack	+ 3–7.7 g proteins/100 g muffin	Yes
Young et al. (2018) (50), Australia	Hospital (*n* = 320) 81 y 53% of women	Protein & Energy	Porridge, sauces, Soups, Desserts	Not specified	Day	Maximum intake: 2030 kcal/d 77 g proteins/d	No
Ziylan et al. (2016) (43), Netherlands	Home (*n* = 120) 71 y 54% of women	Protein & Energy	Sauce, mashed potatoes Creamed spinach	Replacing water with milk powder + cooking cream + Milk powder, cooking cream	Lunch	+ 45–90 kcal/d + 5 g proteins/d	No
Ziylan et al. (2017) (44), Netherlands	Nursing home (*n* = 42) 74 y 67% of women	Protein	Meal	Replacing low protein density ingredients (water, carrots, potatoes, sauce) by high protein density ingredient (milk powder, peas, meat)	Lunch or diner	+ 90 kcal/d + 8 g proteins/d	No

y, years old; d, day; WPI, whey protein isolate; WPC, whey protein concentrate; Wpe, whey permeate.

Overall, 137 DIY fortified recipes were listed: 75 savory and 62 sweet. Among these recipes, 64 were meant to be eaten cold and 67 were meant to be eaten hot (6 can be eaten cold or hot). The food matrices included desserts (*n* = 20 articles; mousse, pie, muffin, cake, biscuit, ice-cream…), meat and fish dishes (*n* = 18; meatball, chicken sticks, marinated duck, baked salmon…), side dishes (*n* = 17; purée, sautéed vegetables), dairy products (*n* = 17; milk, yoghurt, cream), soups (*n* = 14), carbohydrate-based dishes (*n* = 14; oatmeal, cereal, risotto, pancake), beverages (*n* = 9; fruit juice, tea), sauces (*n* = 9), breads (*n* = 8), fruits (*n* = 7; compote/purée, salad, smoothie), eggs dishes (*n* = 3; omelet) and pulse-based dishes (*n* = 1). It is interesting to note that food matrices included both liquids (milk, soup, fruit juice…), semi-liquid foods (purée, yoghurt…) and solid foods (cake, chicken sticks, bread). There was a large variability in the number of matrices used for fortification in the articles. Twelve articles used one only matrix category to be fortified (33–36, 39, 40, 46, 49, 52, 54, 75, 77). Munk et al. (66) developed 36 fortified dishes in collaboration with dietitians, chefs and patients from a hospital. These dishes covered a large range of different food types (soup, meat and fish dishes, vegetable dishes, bread, dessert, beverages).

**Figure 2:**
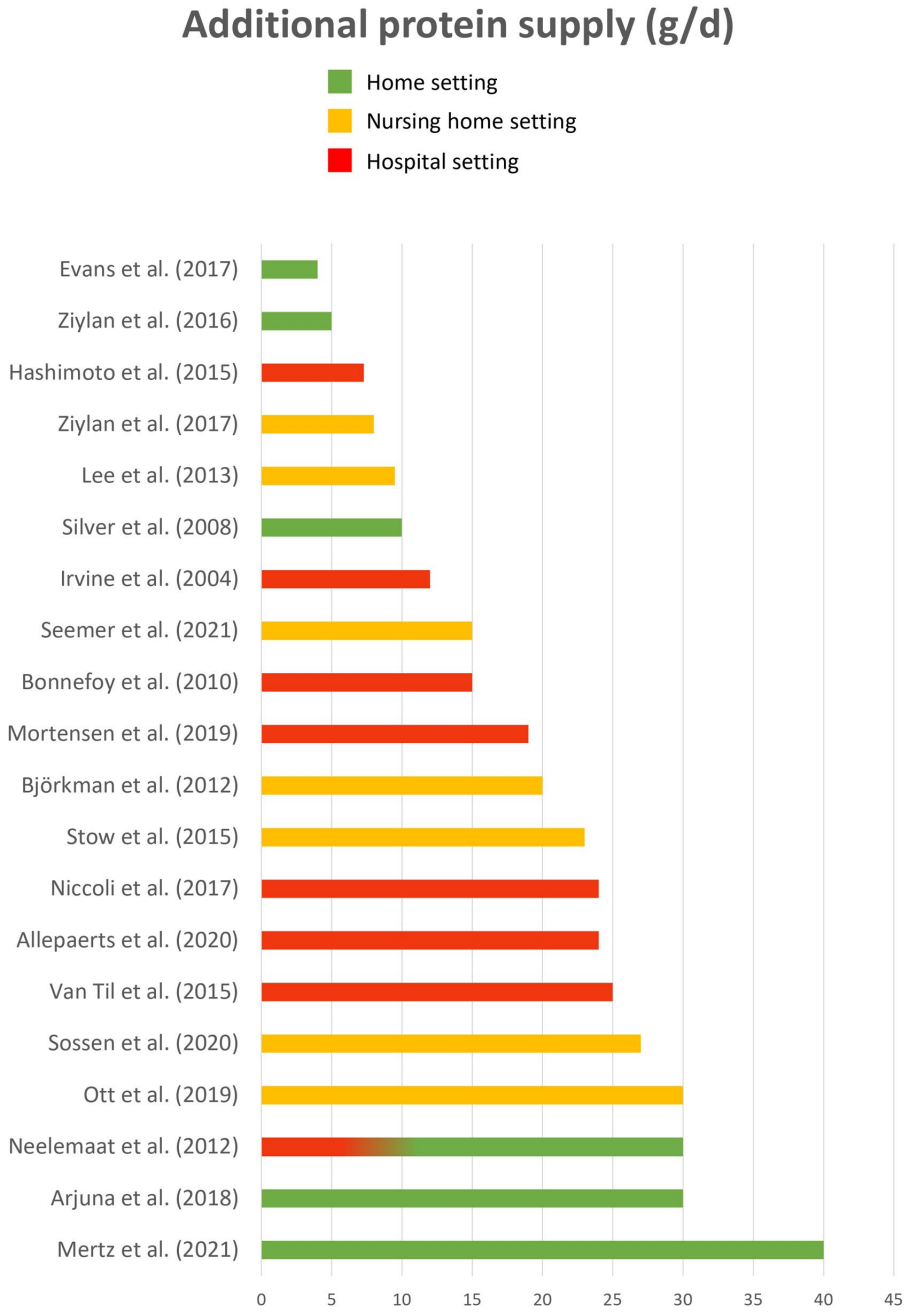
Additional protein load (g/d).

**Figure 3:**
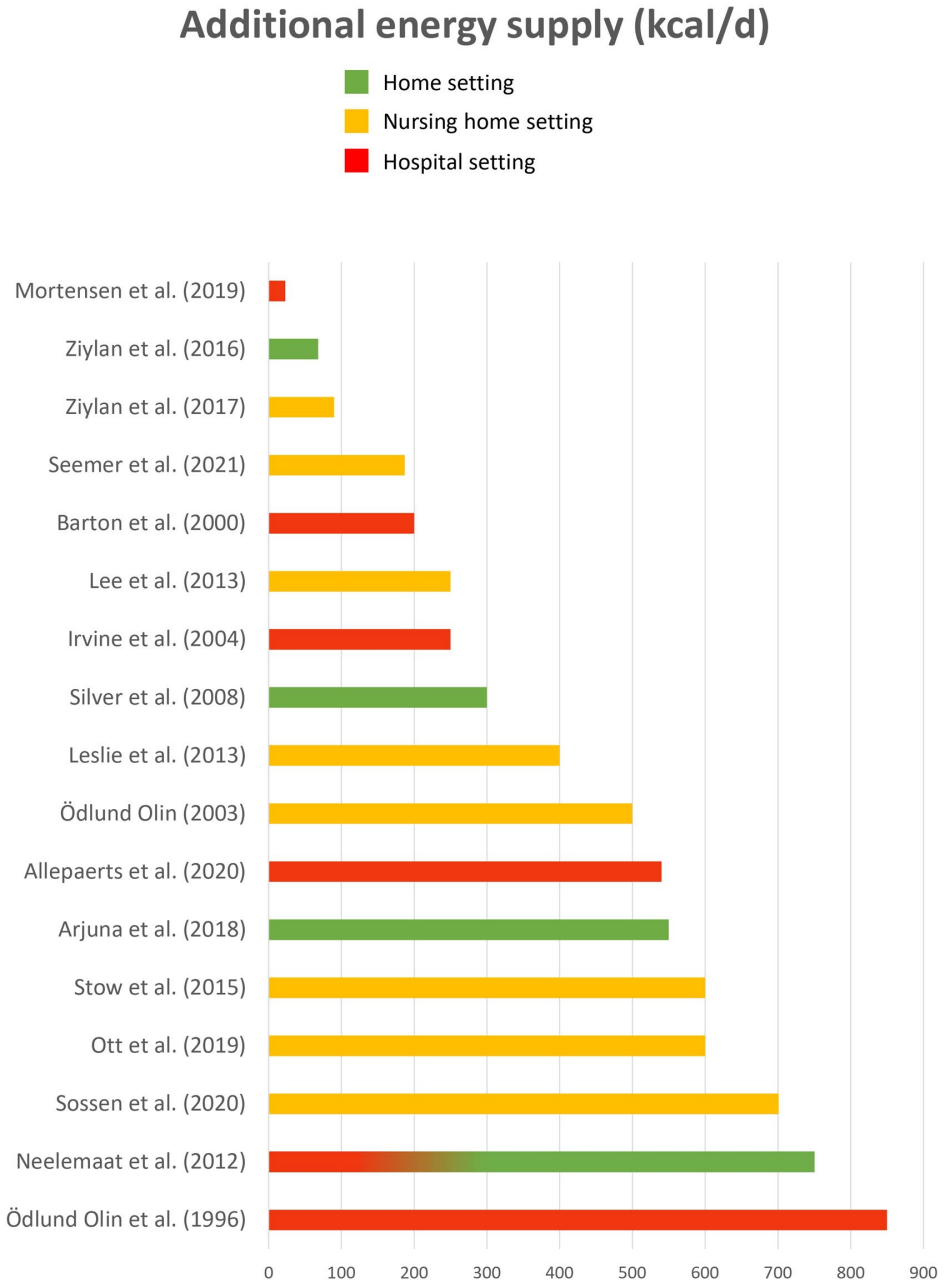
Additional energy load (kcal/d).

Twenty different fortificants were identified across all the studies, including 10 regular food ingredients and 10 macronutrient isolates or concentrates. Four articles (38, 45, 59, 66) did not provide enough details about fortificants (“high fat dairy food,” “dairy,” “non-dairy substitute,” “natural energy-dense ingredient,” “protein powder,” “soy origin”), thus they could not be classified. Seven fortificants targeted energy fortification, 8 targeted protein fortification and 5 targeted both. Most of the fortificants were powdered (*n* = 11). Other fortificants were solid (*n* = 4), semi-liquid (*n* = 3) or liquid (*n* = 2). Energy fortificants included cream (*n* = 20 articles), butter/margarine (*n* = 13), oils (*n* = 10), carbohydrates (*n* = 7), hydrolyzed starch (*n* = 1), mayonnaise (*n* = 1) and maize (*n* = 1). Protein fortificants included whey protein (*n* = 15 articles), protein concentrates/isolates (*n* = 5; Protifar, Hyperprotidine, L-Carnitine…), soy (*n* = 3), pea (*n* = 2), meat (*n* = 2), collagen (*n* = 1), casein (*n* = 1), and gelatine (*n* = 1). Energy and protein fortificants included milk powder (*n* = 10), cheese (*n* = 7), milk (*n* = 5), eggs (*n* = 3) and almonds (*n* = 3). Finally, Figures 2, 3 illustrate the wide variability regarding the additional load of energy and protein provided by fortified food across the studies. This additional load varies from 23 to 850 kcal / day for energy (M = 403; SE = 62) and from 4 to 40 g / day for protein (M = 19; SE = 2).

### 3.4. Assessment of DIY fortified foods acceptability

Thirteen studies have assessed consumer acceptability for DIY fortified foods (Table 3). All these studies conducted the acceptability evaluation with older people except for one (71), who asked nursing home staff to provide feedback on product acceptance based on residents’ observation. Six articles (33, 34, 43, 44, 52, 59) used liking scales to assess product acceptance while the others only collected qualitative data through interviews, focus groups or an acceptability survey. However, most of the articles do not provide enough information about the methodology used to assess acceptability and/or about the results. In most of the articles (*n* = 10/13), acceptability was only a secondary outcome while nutrition was the first one. In these studies, acceptability tests were usually conducted with the same sample as the one recruited for nutritional assessment (the whole sample in 6 articles; a smaller sub-sample in 2 articles). Three articles (33–35) were dedicated to assessing acceptability of DIY fortified foods versus regular foods.

**Table 3:** DIY fortified food acceptability assessment.

Author(s) (year), Country	Panel	Products	Tests/outcomes	Main results
Norton et al. (2020) (33), UK	Home (*n* = 32) 75 y 56% of women	FF vs. SF products: Cupcakes	Liking scale Free comment	FF significantly reduced overall liking compared to SF. Generally positive comments relating to flavor of both FF and SF. FF resulted in a greater number of negative comments related to texture compared to SF.
	Home (*n* = 42) 74 y 55% of women	FF vs. SF products: Cakes and biscuits	Liking scale Free comment Preference test	FF significantly reduced overall liking compared to SF. Generally positive feedbacks for SF while negative feedbacks were generated for FF related to both flavor and texture.
Tsikritzi et al. (2015) (34), UK	Home (*n* = 67) 71 y	FF vs. SF products: Tomato sauce and gravy	Liking scale	Tomato sauce: 2 FF were significantly more liked than SF, while 1 FF was not different from SF. Gravy: FF and SF not different.
Wendin et al. (2017) (35), Sweden	Home (*n* = 7) 60–69 y 71% of women	FF vs. SF products: Muffin	Focus group	SF significantly more liked and accepted than the FF versions.
Arjuna et al. (2018) (48), Australia	Home with HDM (*n* = 29) 83 y 55% of women	FF and SF products	Acceptability survey	FF and SF not compared. For both FF and SF, 50% of participant were (very) satisfied, 16% were unsure and 33% dissatisfied.
Beelen et al. (2017a) (45), Netherlands	Hospital and nursing home (*n* = 22) 83 y 59% of women	FF and SF products: Bread, soup, fruit juice, mashed potatoes	Free comment	Results not provided.
Castellanos et al. (2009) (59), USA	Nursing home (*n* = 21 subjects different from the main study)	FF vs. SF products: Oatmeal, soup, start dish	Hedonic test	FF and SF not different. (data not shown)
Munk et al. (2013) (66), Denmark	Hospital (*n* = 11 subjects different from the main study)	FF menu	Focus group Tasting session	Results not provided.
Nykänen et al. (2019) (52), Finland	Home with home care (*n* = 85) 83 y 72% of women	FF products: Snack	Liking scale	Participants reported acceptability with the product’s taste. (data not shown)
Ott et al. (2019) (71), Germany	Nursing staff from the nursing home	FF menu	Nursing staff’s feedback based on residents’ observation	Enhanced appetite and pleasure with eating were described for 5 residents, whereas 1 did not like the food.
Silver et al. (2008) (37), USA	Older foodservice staff and registered dietitian from the study	FF vs. SF products	Tasting session	Results not provided.
Stelten et al. (2015) (75), Netherlands	Hospital (*n* = 47) 80 y 55% of women	FF vs. SF products: bread and drinking yoghurt	Acceptability survey	For both FF and SF, majority of participants were neutral/positive (77% for bread, 87% for drinking yoghurt) about the taste of the products with no differences between FF and SF.
Ziylan et al. (2016) (43), Netherlands	Home (*n* = 120) 71 y 54% of women	FF vs. SF products: beef and chicken meal	Liking scale	No differences between FF and SF products. Overall liking varied between 5.4 to 6.0.
Ziylan et al. (2017) (44), Netherlands	Nursing home (*n* = 42) 74 y 67% of women	FF vs. SF products: meals and bread	Liking scale Acceptability	No differences about overall evaluation of products. Both FF and SF meals were score 7.7.

y, year old; FF, fortified foods; SF, standard foods.

Seven articles provided results on comparison between DIY fortified and regular foods. Among them, 4 articles (37, 43, 44, 59, 75) reported no significant difference in acceptability when comparing fortified and regular foods while 2 articles (33, 35) reported that fortified foods were less appreciated than regular food. Only one article reported that some fortified foods were more appreciated than regular food, but it depended on the nature of the fortificant added to the food (34). In fact, tomato sauce fortified with cream or with a mix of whey protein and maltodextrin were more liked than regular tomato sauce, but tomato sauce fortified with butter was less liked than regular tomato sauce. Wendin et al. (35) also showed some difference between foods fortified with different fortificants: the regular muffin was more liked than the muffin fortified with almond flour, which was more liked than the whey muffin, itself more liked than the soy muffin.

### 3.5. Assessment of the nutritional impact of DIY food-based fortification

Forty studies assessed the impact of diet enrichment including DIY food-based fortification on nutritional outcomes (food and/or nutrient intakes, nutritional status or body weight) compared to a standard diet (Table 4). Among these studies, 3 combined DIY food-based and diet-based fortification (i.e., modifying the diet by adding nutritionally rich foods), 6 combined food-based fortification and fortified foods marketed by the Food Industry, 1 combined food-based fortification and Oral Nutritional Supplements (ONS), and 2 combined food-based fortification, diet-based fortification and ONS, while 27 studies assessed the impact of DIY food-based fortification alone. Nutritional intake was mainly measured by using dietary record. Nutritional status was mainly assessed by measuring body weight or BMI (20 studies), by using the Mini-Nutritional Assessment Questionnaire [MNA, 8 studies – (39, 48, 49, 52, 57, 72, 73, 77)] or by measuring muscle mass [4 studies – (39, 46, 49, 58)]. A few studies used other indicators such as the Subjective Global Assessment (74) or albumin and pre-albmin (40, 47, 52, 58, 72).

**Table 4:** Comparison between DIY fortified diet and standard diet on nutritional outcomes.

Author(s) (year), Country	Population	Design	Intervention	Control	Fortification type	Additional load of the intervention	Food volume equivalence	Main nutritional outcomes	Main results		
									Evolution in control group^a^	Evolution in intervention group^a^	Intervention *vs* control group^b^
**Arjuna et al. (2018)** (48),**Australia**	**Home with HDM (*n* = 29)** **83 y** **55% of women**	**Between-subject** **parallel** **Longitudinal (3 m)**	**Fortified HDM lunch + dietary counseling**	**Standard HDM lunch + dietary counseling**	**Protein & Energy**	**+ 550 kcal/d** **+ 30 g proteins/d**	**IG = CG**	**Protein intake** **Energy intake** **MNA** **BMI**	**0** **0** **0** **0**	**+** **+** **+** **0**	**Not specified** **Not specified** **Not specified** **Not specified**
**Barton et al. (2000)** (55),**UK**	**Hospital (*n* = 35)** **77 y** **63% of women**	**Within-subject** **cross-over** **Cross-sectional**	**Reduced size fortified diet**	**Standard diet**	**Energy**	**+ 200 kcal/d** **- 5 g proteins/d** **- 20% portion size/meal**	**IG < CG**	**Energy intake**	**NA**	**NA**	**+**
**Beelen et al. (2017a)** (45),**Netherlands**	**Hospital and nursing home (*n* = 22)** **83 y** **59% of women**	**Within-subject** **pre-post** **Longitudinal (10 d)**	**Fortified diet and substitution + Fortified foods/snacks**	**Standard diet**	**Protein**	**Not specified**	**IG > CG**	**Protein intake**	**NA**	**+**	**NA**
**Beelen et al. (2017b)** (56),**Netherlands**	**Hospital (*n* = 147)** **79 y** **55% of women**	**Between-subject** **Cross-sectional**	**Fortified diet + Fortified snacks**	**Standard diet**	**Protein**	**Not specified**	**IG > CG**	**Protein intake**	**NA**	**NA**	**+**
**Beelen et al. (2018)** (57),**Netherlands**	**Home (*n* = 75)** **77 y** **56% of women**	**Between-subject RCT** **Longitudinal (3 m)**	**Fortified foods + Fortified extra options**	**Standard diet**	**Protein**	**Not specified**	**IG > CG**	**Protein intake** **MNA** **BW**	**Not specified** **+** **+**	**Not specified** **+** **0**	**+** **Not specified** **Not specified**
**Beerman et al. (2016)** (42),**Denmark**	**Hospital (*n* = 62)** **69 y** **Not specified**	**Between-subject** **Cross-sectional**	**Fortified breakfast**	**Standard breakfast**	**Protein**	**Maximum intake: 20 g proteins/breakfast**	**Not specified**	**Protein intake**	**NA**	**NA**	**+**
**Björkman et al. (2012)** (39),**Finland**	**Nursing home (*n* = 99)** **84 y** **76% of women**	**Between-subject RCT** **Longitudinal (6 m)**	**Fortified juice during the main meals**	**Standard juice during the main meals**	**Protein**	**+ 20 g proteins/d**	**IG = CG**	**MNA** **BW** **Muscle mass**	**0** **-** **0**	**0** **+** **0**	**0** **+** **0**
**Bonnefoy et al. (2010)** (58),**France**	**Hospital (*n* = 26)** **81 y** **58% of women**	**Between-subject RCT** **Longitudinal (2 w)**	**Fortified lunch and dinner**	**Standard diet**	**Protein**	**+ 11–18 g proteins/d** **(of which 47.5% BCAAs)**	**IG = CG**	**Protein intake** **Muscle mass** **Albumin** **Pre-albumin**	**0** **0** **0** **0**	**0** **0** **0** **0**	**0** **0** **0** **0**
Castellanos et al. (2009) (59), USA	Nursing home (*n* = 26) 87 y 70% of women	Within-subject cross-over Cross-sectional	IG1: Fortified lunch only IG2: Fortified breakfast and lunch	Standard diet	Protein & Energy	+ 4.75 kcal/g food + 0.09 g protein/g food	IG = CG	Protein intake Energy intake	NA	NA	+ (IG1) + (IG2) + (IG1) + (IG2)
**Evans et al. (2017)** (46),**Canada**	**Home (*n* = 41)** **60 y** **64% of women**	**Between-subject RCT** **Longitudinal (2 m)**	**IG1: L-carnitine fortified orange juice** **IG2: L-carnitine combination fortified orange juice**	**Placebo orange juice**	**Protein**	**+ 1.5–6.5 g proteins/d**	**IG = CG**	**Muscle mass**	**0**	**0 (IG1) + (IG2)**	**0 (IG1) + (IG2)**
Gall et al. (1998) (60), UK	Hospital (*n* = 143) 67 y 66% of women	Between-subject Cross-sectional	Fortified lunch and dinner +2 standard snacks	Standard diet	Protein & Energy	+ 966 kcal/d + 22.2 g proteins/d	IG > CG	Protein intake Energy intake	NA	NA	0 +
**Hashimoto et al. (2015)** (61),**Japan**	**Hospital (*n* = 28)** **74 y** **57% of women**	**Between-subject parallel** **Longitudinal (1 m)**	**IG1: Fortified lunch with soy protein** **IG2: Fortified lunch with casein protein**	**Standard diet**	**Protein**	**+ 7.1–7.5 g proteins/d**	**IG = CG**	**BW**	**0**	**0 (IG1) 0 (IG2)**	**0 (IG1) 0 (IG2)**
**Irvine et al. (2004)** (36),**France**	**Hospital (*n* = 12)** **84 y** **33% of women**	**Within-subject cross-over** **Cross-sectional**	**IG1: Standard breakfast + fortified low-protein drink** **IG2: Standard breakfast + fortified high-protein drink**	**Standard breakfast**	**Protein & Energy**	**+ 250 kcal/d** **+ 3.5–20 g proteins/d**	**IG > CG**	**Protein intake** **Energy intake**	**NA**	**NA**	**0 (IG1) + (IG2)** **0 (IG1) 0 (IG2)**
Iuliano et al. (2013) (62), Australia	Nursing home (*n* = 130) 88 y 78% of women	Between-subject RCT Longitudinal (1 m)	Substitution, fortification and additional food items	Standard diet	Protein & Energy	Not specified	IG > CG	Protein intake Energy intake	0 0	+ +	Not specified Not specified
**Lee et al. (2013)** (40),**Taiwan**	**Nursing home (*n* = 83)** **80 y** **58% of women**	**Between-subject RCT** **Longitudinal (6 m)**	**Fortified warm drink snack**	**Warm soup snack**	**Protein**	**+ 250 kcal/d** **+ 9.5 g proteins/d**	**IG = CG**	**BW** **Albumin**	**-** **0**	**0** **+**	**+** **+**
Leslie et al. (2013) (63), UK	Nursing home (*n* = 31) 91 y 88% of women	Between-subject RCT Longitudinal (3 m)	Fortified diet + standard snack	Standard diet	Energy	+ 400 kcal/d	IG > CG	Energy intake BW	0 0	0 +	0 0
**Lorefält et al. (2005)** (64),**Sweden**	**Hospital (*n* = 10)** **82 y** **60% of women**	**Within-subject** **Cross-sectional**	**Reduced size fortified lunch and dinner + 2 standard snacks**	**Standard diet + 2 standard snacks**	**Protein & Energy**	**+ 0 kcal/d** **+ 0 g proteins/d** **– 50% portion size**	**IG < CG**	**Protein intake** **Energy intake**	**NA**	**+** **+**	**NA**
**Mertz et al. (2021)** (65),**Denmark**	**Home (*n* = 184)** **70 y** **46% of women**	**Within-subject** **pre-post** **Longitudinal (12 m)**	**IG1: Fortified whey protein drink** **IG2: Fortified collagen protein drink**	**Standard diet**	**Protein**	**+ 40 g protein/d**	**IG = CG**	**Protein intake** **BW**	**NA**	**+ (IG1) + (IG2)** **0 (IG1) 0 (IG2)**	**NA**
**Mortensen et al. (2019)** (51),**Denmark**	**Hospital (*n* = 92)** **69 y** **56% of women**	**Between-subject** **Cross-sectional**	**Fortified snacks**	**Standard snacks**	**Protein**	**+ 18–27 kcal/d** **+ 15–23 g proteins/d**	**IG = CG**	**Protein intake**	**NA**	**NA**	**+**
**Munk et al. (2013)** (66),**Denmark**	**Hospital (*n* = 79)** **73 y** **75% of women**	**Between-subject** **Cross-sectional**	**Standard diet + fortified small dishes**	**Standard diet**	**Energy**	**Not specified**	**IG > CG**	**Energy intake**	**NA**	**NA**	**0**
**Munk et al. (2014)** (41),**Denmark**	**Hospital (*n* = 78)** **75 y** **58% of women**	**Between-subject RCT** Longitudinal (15 d)*	**Standard diet + Fortified small meals**	**Standard diet**	**Protein & Energy**	**+ 0.6–4.7 kcal/g** **+ 6.1–11.5 g proteins/serving**	**IG > CG**	**Protein intake** **Energy intake** **BW**	**NA** **NA** **0**	**NA** **NA** **0**	**+** **0** **0**
Neelemaat et al. (2012) (67), Netherlands	Hospital + Home (*n* = 150) 75 y 55% of women	Between-subject RCT Longitudinal (3 m)	Individual nutritional care (fortified diet (only during hospital stay), ONS, telephone counseling, vitamin D3)	Standard nutritional care	Protein & Energy	Hospital phase: + 750 kcal/d; + 30 g proteins/d Home phase: + 600 kcal/d; + 24 g proteins/d	IG > CG	Protein intake Energy intake BW	Not specified Not specified Not specified	Not specified Not specified Not specified	+ + +
**Niccoli et al. (2017)** (47),**Canada**	**Hospital (*n* = 47)** **81 y** **68% of women**	**Between-subject RCT** Longitudinal (2026 d)*	**Fortified diet**	**Standard diet**	**Protein**	**+ 24 g proteins/d**	**IG = CG**	**Protein intake** **Albumin** **Pre-albumin**	**NA** **0** **0**	**NA** **0** **0**	**+** **0** **0**
Nykänen et al. (2019) (52), Finland	Home with home care (*n* = 85) 83 y 72% of women	Between-subject RCT Longitudinal (3 m)	Standard diet + Fortified snacks	Standard diet	Energy	+ 272–282 kcal/d + 14.3–14.9 g proteins/d	IG > CG	MNA BMI Albumin Pre-albumin	0 0 - -	+ 0 0 0	+ 0 + 0
**Ödlund Olin (2003)** (69),**Sweden**	**Nursing home (*n* = 35)** **82 y** **52% of women**	**Between-subject parallel** **Longitudinal (17 w)**	**Fortified diet**	**Standard diet**	**Energy**	**+ 500 kcal/d**	**IG = CG**	**Energy intake** **BW**	**0** **0**	**+** **0**	**+** **0**
**Ödlund Olin et al. (1996)** (70),**Sweden**	**Hospital (*n* = 36)** **82 y** **67% of women**	**Within-subject cross-over** **Longitudinal (6 w)**	**Fortified lunch and dinner + fortified snacks**	**Standard diet + regular snacks**	**Energy**	**+ 850 kcal/d**	**IG = CG**	**Energy intake** **BW**	**Not specified** **0**	**Not specified** **+**	**+** **+**
**Ott et al. (2019)** (71),**Germany**	**Nursing home (*n* = 16)** **87 y** **88% of women**	**Within-subject** **pre-post** **Longitudinal (6 w)**	**Fortified textured-modified diet + 1 fortified snack + extra fortified choice**	**Standard texture-modified diet + 3 standard snacks**	**Protein & Energy**	**+ 600 kcal/d** **+ 30 g proteins/d**	**IG = CG**	**Protein intake** **Energy intake** **BW**	**0** **0** **0**	**0** **0** **0**	**+** **+** **+**
**Park et al. (2018)** (49),**Korea**	**Home (*n* = 99)** **77 y** **65% of women**	**Between-subject RCT** **Longitudinal (3 m)**	**IG1: fortified tea to reach 1.2 g proteins/kg/d** **IG2: fortified tea to reach 1.5 g proteins/kg/d**	**Placebo tea to reach 0.8 g proteins/kg/d (in tea)**	**Protein**	**+ 0.4–0.7 g proteins/kg/d**	**IG = CG**	**Protein intake** **MNA** **Muscle mass**	**Not specified** **Not specified** **Not specified**	**Not specified** **Not specified** **Not specified**	**+ (IG1) + (IG2)** **0 (IG1) 0 (IG2)** **0 (IG1) + (IG2)**
Polonen et al. (2017) (72), Finland	Home with home care (*n* = 227) 84 y 71% of women	Between-subject parallel Longitudinal (6 m)	Individual nutritional care (dietary counseling for increasing protein and energy intake, ONS when needed, vitamin D)	Standard nutritional care	Protein & Energy	Not specified	IG > CG	MNA BMI Albumin	0 0 0	+ 0 +	+ 0 +
Seemer et al. (2021) (54), Germany	Nursing home (*n* = 50) 84 y 74% of women	Within-subject pre-post Longitudinal (6 w)	Individualized nutritional intervention (reshaped texture-modified meals and 3 enriched supplements)	Usual nutritional care	Protein	+ 125–470 kcal/d + 1,042 g protein/d	IG > CG	Protein intake BW	NA	+ 0	NA
**Silver et al. (2008)** (37),**USA**	**Home with HDM (*n* = 45)** **84 y** **69% of women**	**Within-subject cross-over** **Cross-sectional**	**Fortified HDM lunch**	**Standard HDM lunch**	**Protein & Energy**	**+ 300 kcal/d** **+ 10 g proteins/serving**	**IG = CG**	**Protein intake** **Energy intake**	**NA**	**NA**	**+** **+**
**Smoliner et al. (2008)** (73),**Germany**	**Nursing home (*n* = 52)** **83 y** **73% of women**	**Between-subject RCT** **Longitudinal (3 m)**	**Fortified soup and sauce + 2 fortified snacks**	**Standard diet**	**Protein & Energy**	**Not specified**	**IG > CG**	**Protein intake** **Energy intake** **MNA** **BW**	**NA** **NA** **+** **+**	**NA** **NA** **+** **+**	**+** **0** **0** **0**
**Sossen et al. (2020)** (74),**Australia**	**Nursing home (*n* = 122)** **88 y** **76% of women**	**Within-subject** **pre-post** **Longitudinal (6 m)**	**Fortified diet**	**Standard diet**	**Protein & Energy**	**+ 701 kcal/d** **+ 27 g proteins/d**	**IG = CG**	**SGA** **BW**	**NA**	**0** **0**	**NA**
Starke et al. (2011) (38), Switzerland	Hospital (*n* = 132) 73 y Not specified	Between-subject RCT Longitudinal (16 d)*	Individual nutritional care (detailed nutritional assessment, individual food supply, fortified meals, ONS, in between-meals snacks)	Standard nutritional care (ONS, Nutritional therapy)	Protein & Energy	Not specified	IG > CG	Protein intake Energy intake BW	NA NA -	NA NA 0	+ + +
Stelten et al. (2015) (75), Netherlands	Hospital (*n* = 47) 80 y 55% of women	Between-subject Cross-sectional	Fortified bread and drinking yoghurt	Standard diet	Protein	+ 16 g proteins/serving (*ad libitum*)	IG = CG	Protein intake	NA	NA	+
**Stow et al. (2015)** (76),**UK**	**Nursing home (*n* = 67)** **Not specified** **82% of women**	**Between-subject RCT** **Longitudinal (6 m)**	**Standard diet + Fortified meals**	**Standard diet**	**Protein & Energy**	**+ 600 kcal/d** **+ 20–25 g proteins/d**	**IG > CG**	**Protein intake** **Energy intake** **BW**	**Not specified** **Not specified** **Not specified**	**Not specified** **Not specified** **Not specified**	**0 (M3) 0 (M6)** **+ (M3) + (M6)** **+ (M3) 0 (M6)**
Van Til et al. (2015) (77), Netherlands	Hospital (*n* = 34) 78 y 68% of women	Between-subject RCT Longitudinal (3 w)	Fortified bread and drinking yoghurt	Standard diet	Protein	+ 17 kcal/serving + 8 g proteins/serving	IG = CG	Protein intake MNA BW	NA 0 0	NA 0 0	+ 0 0
Young et al. (2018) (50), Australia	Hospital (*n* = 320) 81 y 53% of women	Between-subject Cross-sectional	Fortified diet + Standard snacks + ONS	Standard diet + Standard snacks + ONS	Protein & Energy	Maximum intake: 2030 kcal/d 77 g proteins/d	IG = CG	Protein intake Energy intake	NA	NA	+ +
**Ziylan et al. (2016)** (43),**Netherlands**	**Home (*n* = 120)** **71 y** **54% of women**	**Within-subject cross-over** **Cross-sectional**	**Fortified beef meal and chicken meal**	**Standard beef meal and chicken meal**	**Protein & Energy**	**+ 45–90 kcal/d** **+ 5 g proteins/d**	**IG = CG**	**Protein intake** **Energy intake**	**NA**	**NA**	**+** **+**
Ziylan et al. (2017) (44), Netherlands	Nursing home (*n* = 42) 74 y 67% of women	Between-subject RCT Longitudinal (2 w)	Fortified bread and meals	Standard bread and meals	Protein	+ 90 kcal/d + 8 g proteins/d	IG = CG	Protein intake	Not specified	Not specified	+

y, year old; d, days; m, months; w, weeks; RCT, randomized controlled trial; HDM, home-delivery meal; FF, fortified group; SF, standard group; BMI, body mass index; BW, body weight; MNA, Mini Nutritional Assessment; SGA, Subjective Global Assessment; NA, not applicable; +, significant increase; −, significant decrease; 0, no significant differences. Articles with outcomes labeled “Not specified” did not show statistical value of p test for outcomes concerned. **Bold:** study that assess the impact of DIY fortification alone. *Mean length of stay from admission to discharge. ^a^Comparison with baseline and follow-up; ^b^Comparison between control group and fortified group (in this column “+” means that results are significantly higher for fortified group compared to control group).

When all the studies are considered, results highlight that provided protein-fortified foods led to a significant increase in protein intake (26 studies over 29) and that provided energy-fortified led to a significant increase in energy intake (15 studies over 20). Only a few studies showed a significant impact of DIY fortification on nutritional status compared to regular food offer: 3 out 8 observed a significant impact on MNA score, 7 out 20 observed a significant impact on body weight or BMI and 2 out 4 observed a significant impact on muscle mass. None observed a negative impact.

When only the studies which assessed the impact of DIY fortification alone are considered (in bold in the Table 4), results still highlight that provided protein-fortified foods led to a significant increase in protein intake (16 studies over 18) and that provided energy-fortified led to a significant increase in energy intake (9 studies over 13). Only a few studies showed a significant impact of DIY fortification on nutritional status compared to regular food offer: 1 out 5 observed a significant impact on MNA score, 4 out 13 observed a significant impact on body weight or BMI and 1 out 3 observed a significant impact on muscle mass.

### 3.6. Comparison of DIY food-based fortification with other alternatives

Seven studies evaluated two DIY food-based fortification strategies with either different energy/protein loads (36, 49, 59), different fortificants (46, 61, 65) or different portion sizes (43). Four studies compared DIY food-based fortification with another alternative such as ONS (76), (74), adding high-energy and/or high-protein food items to the menu (55), or increased staff assistance to older people during mealtime (50) (Table 5). However, very few studies have produced statistics to compare the different options. Not surprisingly, higher energy/protein loads are associated with higher energy/protein intake (36, 49). However, there was no significant difference between the 1.2 and the 1.5 g of protein / kg of body weight / day in the evolution of nutritional status and muscle mass over the 12-week intervention (49). In Ziylan et al. (43), the reduced-size enriched chicken meal led to a significantly higher energy intake than the normal-size meal. However, the difference in intake was rather small and no impact of portion size was observed for the enriched beef meals. In Evans et al. (46), a combination of three amino acids significantly improved muscle mass over 2 months while no change was observed when a single amino acid was used to fortify the orange juice. Stow et al. (76) observed no difference between food-based fortification and ONS while Sossen et al. (74) observed a slight advantage for DIY food-based fortification compared to ONS. Energy and protein intakes were higher with DIY fortification than with ONS, and body weight was stable with DIY fortification whereas it decreased with ONS during the 6 months of follow-up. Finally, providing DIY fortified food led to higher energy and protein intake when compared with improving staff assistance to older people during mealtime (50).

**Table 5:** Comparison between DIY fortification and other alternatives.

Author(s) (year), Country	Population	Design	Fortified group (FG)	Alternative group (AG)	Fortification type	Food volume equivalence	Main nutritional outcomes	Main results
Barton et al. (2000) (55), UK	Hospital (*n* = 35) 77 y 63% of women	Within-subject cross-over Cross-sectional	Fortified breakfast	Breakfast with additional energy and protein foods	Energy	Not specified	Nutritional intake	FG vs. AG not compared.
Castellanos et al. (2009) (59), USA	Nursing home (*n* = 26) 87 y 70% of women	Within-subject cross-over Cross-sectional	Fortified lunch only	Fortified breakfast and lunch	Protein & Energy	FG = AG	Nutritional intake	FG vs. AG not compared.
Evans et al. (2017) (46), Canada	Home (*n* = 41) 60 y 64% of women	Between-subject RCT Longitudinal (2 m)	Fortified orange juice with carnitine	Fortified orange juice with carnitine, creatine and leucine	Protein	FG = AG	Muscle mass	FG vs. AG not compared. FG did not change while AG significantly increased over time.
Hashimoto et al. (2015) (61), Japan	Hospital (*n* = 28) 74 y 57% of women	Between-subject parallel Longitudinal (1 m)	Fortified lunch with soy	Fortified lunch with casein	Protein	FG = AG	BW	FG vs. AG not compared. FG and AG did not change over time.
Irvine et al. (2004) (36), France	Hospital (*n* = 12) 84 y 33% of women	Within-subject cross-over Cross-sectional	Fortified low-protein drink	Fortified high-protein drink	Protein & Energy	FG = AG	Nutritional intake	FG < AG
Mertz et al. (2021) (65), Denmark	Home (*n* = 184) 70 y 46% of women	Between-subject RCT Longitudinal (12 m)	Fortified whey protein drink	Fortified collagen protein drink	Protein	FG = AG	Nutritional intake Body weight	FG vs. AG not compared. FG and AG significantly increased over time. FG vs. AG not compared. FG and AG did not change over time.
Park et al. (2018) (49), Korea	Home (*n* = 99) 77 y 65% of women	Between-subject RCT Longitudinal (3 m)	Fortified tea with 1.2 g proteins/kg/d	Fortified tea with 1.5 g proteins/kg/d	Protein	FG = AG	Nutritional intake Nutritional status Muscle mass	FG < AG. No significant difference between FG and AG. No significant difference between FG and AG.
Sossen et al. (2020) (74), Australia	Nursing home (*n* = 122) 88 y 76% of women	Between-subject parallel Longitudinal (6 m)	Fortified meals	ONS	Protein & Energy	Not specified	Nutrition intake Nutritional status BW	FG > AG FG vs. AG not compared. FG and AG did not change over time. FG vs. AG not compared. FG did not change and AG significantly decreased over time.
Stow et al. (2015) (76), UK	Nursing home (*n* = 67) Not specified 82% of women	Between-subject RCT Longitudinal (6 m)	Fortified meals	ONS	Protein & Energy	Not specified	Nutritional intake BW	No significant difference between FG and AG. No significant difference between FG and AG.
Young et al. (2018) (50), Australia	Hospital (*n* = 320) 81 y 53% of women	Between-subject Cross-sectional	Fortified meals	Assistance during meals	Protein & Energy	Not specified	Nutritional intake	FG > AG
Ziylan et al. (2016) (43), Netherlands	Home (*n* = 120) 71 y 54% of women	Within-subject cross-over Cross-sectional	Normal size enriched meal	Reduced size enriched meal	Protein & Energy	FG > AG	Nutritional intake	Beef meal: No significant difference between FG and AG. Chicken meal: AG > FG

y, year old; RCT, randomized controlled trial; m, months; FG, fortified group; AG, alternative group; NS, non-significant; ONS, oral nutritional supplement; BW, body weight; BMI, body mass index; RDA, recommended daily allowance.

## 4. Discussion

### 4.1. Originality/value of the present review

A survey of the literature allowed the identification of five systematic literature reviews close to the topic of the present review (21–24, 30). Firstly, the systematic review of Trabal and Farran-Codina (23) investigated whether, compared to a standard diet, DIY food-based fortification with regular ingredients and/or powdered modules could improve energy and protein intake in older adults in hospital settings, long-care facilities or home settings. This review included 9 articles. The authors concluded that DIY fortification is a valid intervention for improving energy intake in older adults yet there was insufficient evidence for protein intake, nutritional status and body weight. Secondly, Morilla-Herrera et al. (21) targeted all studies related to DIY food-based fortification with macronutrients to prevent the risk of malnutrition in older patients receiving hospital services for acute or chronic disease, in older people living in nursing homes and in older people with home-care. This review encompassed 7 articles, and the meta-analysis highlighted that DIY food-based fortification yields positive results in the total amount of ingested calories and protein. Thirdly, Douglas et al. (22) aimed to evaluate the effect of DIY fortification with regular food ingredients (excluding protein powders) on energy and protein intake compared to standard diet among adults aged 60 and more in acute-care hospitals, long-term care settings or living at home. Ten articles were included. This review suggested that DIY fortification was effective in increasing energy and protein intake among older individuals. Fourthly, the systematic review by Mills et al. (24) explored the evidence for the use of energy and/or protein dense meals (DIY food-based fortification) or additional snacks (diet-based fortification) to increase the dietary energy and protein intake of adults older than 60 in hospital or rehabilitation facilities. Ten articles were identified. Authors reported that when compared with usual nutritional care, DIY fortification could be an effective, well-tolerated and cost-effective intervention to improve dietary intake among hospitalized patients. Finally, Sossen et al. (30) investigated the effect of food-based and diet-based fortification on energy and protein intake compared to any/no nutritional strategy in residents living in nursing homes. Sixteen articles were included. The results of the meta-analysis showed that fortified menus may significantly increase energy and protein intakes compared with standard menus.

The present review retrieved 44 articles that tested DIY food-based fortification in people over the age of 65. This review differs from previous reviews in the following respects. Firstly, we focused the review on DIY food-based fortification, i.e., the addition of regular food ingredients or macronutrient extracts into conventional food matrices to increase energy and protein content in the final dishes. Douglas et al. (22) considered only culinary ingredients. Mills et al. and Sossen et al. (24, 30) considered both food-based fortification and diet-based fortification via the addition of supplementary conventional foods like snacks to participants’ diets. Second, we considered all living settings, i.e., at home, with or without assistance, institutions and hospitals [Morilla-Herrera et al. (21) only considered dependent older people]. Thirdly, we considered not only nutritional outcomes but also acceptability outcomes. In addition, we used a wide range of keywords to account for the lack of consensual terminology regarding the concept of DIY food-based fortification (Supplementary material). This allowed us to identify a much larger number of articles than in previous reviews.

### 4.2. Description of DIY fortified recipes

A wide variety of DIY fortified recipes were extracted from this review, including liquid (35% of the recipes), semi-solid (17%) and solid food matrices (48%). However, the quality evaluation of the articles highlighted the lack of information provided by the authors on the description of fortified recipes. Only 8 articles provided sufficient information for a third party to reproduce the same fortified recipes as used in the articles. In order to identify efficient DIY fortified solutions, it is essential that in future articles provide a detailed description of the fortified recipes, including the nature of food matrices and fortificants, final energy and protein concentration, additional nutrient load provided by the fortified food compared to the standard food, consumption time, and portion size. From the information collected, energy fortification is mainly achieved through the use of fats and dairy products (cream, butter, oil) while protein fortification is mainly achieved through protein extracts. Such products are usually in powder form (‘protein powders’) and proved to have varied applications and uses within food processing as well as high nutritional and functional value (68). The present review showed that the protein products used in fortified recipes were mainly derived from animal sources (85% of the recipes), especially from milk (67% of the recipes), and to a lesser extent from plant sources (15% of the recipes). Animal-derived proteins are more readily digestible and effective in muscle protein synthesis than plant derived proteins (78).

### 4.3. Evaluation of DIY food-based fortification solutions

Results suggest that food-based fortification is an effective strategy to improve energy and/or protein intake. This trend is observed whether all the studies – including the ones that combined DIY fortification with other strategies (i.e., providing ONS, additional food items, fortified foods from Food Industry) or whether only the studies which assessed the impact of DIY fortification alone are considered. In other words, DIY fortification seems to be an effective strategy to improve nutritional intake, whether used alone or combined with other enrichment strategies. However, no strong evidence is observed regarding the impact of DIY fortification to improve the nutritional status (e.g., MNA score, body weight, muscle mass).

It should be noted that providing fortified food was not necessarily enough to get participants to meet the recommended nutritional allowance (50, 55, 60, 75). For instance, in Stelten et al. (75), 64% of the fortified group did not reach the threshold of 1.2 g protein/kg of body weight/day. This raises the question of the need for new fortification solutions with higher levels of energy and protein content. In addition, consuming fortified foods throughout the various meals of the day may be more efficient than consuming fortified foods only once per day. For instance, Castellanos et al. (59) reported higher energy intake when both breakfast and lunch were fortified than when only lunch was fortified, but they did not carry out statistical analysis to compare these two conditions.

Besides the relatively large number of studies that have tested the impact of DIY food-based fortification on nutritional outcomes, very few studies have looked at the acceptability of DIY fortified food. Only 10 of the 41 nutrition-related articles reported an evaluation of the acceptability of DIY fortified foods and only 3 of the 44 articles included in this review were completely devoted to the assessment of acceptability of DIY fortified food. Unsurprisingly, the quality of the acceptability studies is much better in the articles focused only on this outcome than in the articles that conducted an acceptability study alongside a nutritional study. In the latter, the sample size is often insufficient, the methods are often qualitative and the results are often imprecise and incomplete. In addition, the people who assess the acceptability of fortified food are sometimes different from the end-users [e.g., the fortified foods are tasted by the staff (37)]. Overall, the results tend to show that DIY fortified foods are equally or less appreciated than standard foods – never more. However, before drawing any final conclusions, there is a need to carry out further acceptability studies with a higher quality, taking into account the good practices and the norms of sensory evaluation (79, 80). Indeed, fortified foods should not only be good from a nutritional point of view, but also “good to eat” to ensure that they are actually consumed by the target population. Furthermore, it would be worthwhile to optimize the sensory quality of fortified foods by recruiting older adults in tasting panels. Fortification improvement based on older people’s feedback led to increased food intake in nursing homes (81, 84).

### 4.4. Limitations and strengths of the present SLR

The strength of this paper is its reliable literature search, with a complete overview of nutritional and acceptability issues for fortified food targeting older people. Given the lack of a consensual definition of the concept of food-based fortification, we have used a broad set of keywords to retrieve articles of interest. The limitations of the present literature review are the following: the literature search strategy did not include trial registries, nor grey literature, and it was restricted to English papers. There are two discrepancies between the present method and the one published before the review was carried out. In the published method, we considered including papers published in both English and French (the authors’ native language), but papers in French were ultimately excluded in order to avoid a language bias in the literature search. In addition, in the published method, we considered including papers related to micronutrients fortification, but ultimately focused the scope of the present review on macronutrient fortification, otherwise the scope of the review would have been too broad. Finally, a limitation lies in the fact that it was not always easy to determine whether the products used in the nutritional interventions were a DIY fortified food, a fortified food marketed by the Food Industry or an ONS. For instance, we excluded the studies where enrichment consisted of providing participants with a sachet of nutrient constituents to be dissolved in water [for instance (82, 83)]. Indeed, dissolving a sachet of powder in water is more like taking a drug than having a drink. Conversely, all the interventions consisting of adding a nutrient-dense ingredient to a food matrix were included, even when the fortificant was very specific [for example, branched chain amino acids powder (58), L-carnitine (46)]. However, the question arises as to the accessibility of this type of fortificant to the end-user in real life.

## 5. Conclusion

The present systematic literature review highlighted that, compared to a standard diet, DIY food-based fortification – i.e., incorporating ingredients of nutritional interest into commonly consumed foods – is a valuable strategy for increasing energy and protein intake in older people. However, no strong evidence was observed regarding the impact of DIY fortification to improve the nutritional status (i.e., MNA score, body weight, muscle mass). In addition, further research is needed to better assess the acceptability of this strategy among end-users. Given the limitations of the studies included in this systematic review, we put forward four recommendations for future research. First, we emphasize the need to develop a consistent definition of DIY food-based fortification that clearly distinguishes this strategy from other enrichment strategies such as the consumption of ONS or fortified food from food industry. Second, it would be useful for studies to better describe the recipes used for DIY fortification. This information would result in a better understanding of the factors that maximize the impact of food-based fortification on nutritional outcomes. Third, it would be relevant to systematically assess the acceptability of DIY fortified foods in addition to the nutritional outcomes. This should be done by implementing consumer tests that respect the good practices and the recommendations defined in sensory evaluation for such tests (sample size, methods…). To achieve this, it is essential to encourage more pluri-disciplinary research projects involving experts in nutrition, sensory evaluation and food technology. Fourth, we encourage researchers to further compare the impact of food-based fortification with other enrichment strategies, and in particular ONS, in order to better decipher the impact of each of these strategies in tackling undernutrition in the older people. Finally, future research should also study how to promote DIY food fortification among the older people, their caregivers, as well as among catering and health professionals. Indeed, despite this strategy has proved effective in sustaining caloric and protein intake in older people, it remains largely unknown and underused. Several dissemination strategies could be considered. A first one could be the development and the diffusion of DIY fortified recipes booklets. Such booklets should indicate the amount of protein provided by each portion. These booklets would also need to be co-created with end-users, to ensure the feasibility and acceptability of the recipes in the field, considering various settings (home cooking, home-delivery meals, nursing home, hospital). A second dissemination strategy could be the organization of therapeutic workshops at hospital discharge or in day hospital, bringing together dieticians, chefs and older people to promote DIY food fortification. However, from a more global perspective, public policies are needed to raise awareness of the nutritional needs of the older people. These policies must combine information and tools to maintain adequate energy and protein intakes, in order to prevent undernutrition in the older population.

## Data availability statement

The original contributions presented in the study are included in the article/Supplementary material, further inquiries can be directed to the corresponding author/s.

## Author contributions

AG: methodology, investigation, formal analysis, and writing – original draft. MP: methodology, investigation, formal analysis, and writing – review and editing. VW-D: conceptualization and writing – review and editing. CS-R: conceptualization, methodology, formal analysis, writing – original draft, and funding acquisition. All authors contributed to the article and approved the submitted version.

## Footnotes

^1^https://hal.archives-ouvertes.fr/hal-03180038
