# Supplementary material for: Impact of food-based fortification on nutritional outcomes and acceptability in older adults: systematic literature review
Source: Front Nutr. 2023 Oct 27;10:1232502. doi: 10.3389/fnut.2023.1232502 (PMC10641731; doi:10.3389/fnut.2023.1232502)
Supplement: Supplementary file 1 [file Data_Sheet_1.docx]

# Supplementary Files

Search strategy in Web of Science, PubMed and Scopus. (For PubMed, MeSH terms are in bold).

**Old people**

"**aged**" OR "elderly" OR "older" OR "senior" OR "aging" OR "ageing"

AND

**Food-based fortification**

"**food, fortified**" OR "enriched food" OR "enriched foods" OR "food enrichment" OR "diet enrichment" OR "enriched diet" OR "enriched diets" OR "food fortification" OR "supplemented food" OR "supplemented diet" OR "supplemented diets" OR "food supplementation" OR "diet supplementation" OR "additional food" OR "additional foods" OR "added food" OR "fortified drink" OR "fortified beverage" OR "enriched drink" OR "enriched beverage" OR "enriched beverages" OR "dense food" OR "dense foods" OR "dense diet" OR "dense diets" OR "**food, formulated**" OR "formulated food" OR "fortified foods" OR "dietary enrichment"

AND

**Outcomes**

***Nutritional intake***: "eating" OR "food intake" OR "dietary intake" OR "feed intake" OR "food consumption" OR "diet pattern" OR "dietary pattern" OR "nutritional intake" OR "protein intake" OR "energy intake" OR "**nutritional requirement**" OR "nutritional requirements" OR "dietary protein" OR "dietary proteins" OR "feeding" OR "**feeding behavior**"

OR

***Nutritional status:*** "nutritional status" OR "**body weight**" OR "weight" OR "Body Mass Index" OR "BMI" OR "muscle mass" OR "**malnutrition**" OR "undernutrition" OR "undernourished" OR "malnourished" OR "appetite" OR "sarcopenia" OR "**frail elderly**" OR "frailty" OR "frail" OR "**elder nutritional physiological phenomena**"

OR

***Acceptability:*** "acceptability" OR "preference" OR "preferences" OR "liking" OR "acceptance" OR "palatability" OR "palatable" OR "expectation" OR "expectations"

| **Section/topic** | **#** | **Checklist item** | **Reported on page #** |
| --- | --- | --- | --- |
| **TITLE** | | |  |
| Title | 1 | Identify the report as a systematic review, meta-analysis, or both. | 1 |
| **ABSTRACT** | | |  |
| Structured summary | 2 | Provide a structured summary including, as applicable: background; objectives; data sources; study eligibility criteria, participants, and interventions; study appraisal and synthesis methods; results; limitations; conclusions and implications of key findings; systematic review registration number. | 1 |
| **INTRODUCTION** | | |  |
| Rationale | 3 | Describe the rationale for the review in the context of what is already known. | 2 |
| Objectives | 4 | Provide an explicit statement of questions being addressed with reference to participants, interventions, comparisons, outcomes, and study design (PICOS). | 2 |
| **METHODS** | | |  |
| Protocol and registration | 5 | Indicate if a review protocol exists, if and where it can be accessed (e.g., Web address), and, if available, provide registration information including registration number. | 2 |
| Eligibility criteria | 6 | Specify study characteristics (e.g., PICOS, length of follow-up) and report characteristics (e.g., years considered, language, publication status) used as criteria for eligibility, giving rationale. | 3 |
| Information sources | 7 | Describe all information sources (e.g., databases with dates of coverage, contact with study authors to identify additional studies) in the search and date last searched. | 3 |
| Search | 8 | Present full electronic search strategy for at least one database, including any limits used, such that it could be repeated. | 3 |
| Study selection | 9 | State the process for selecting studies (i.e., screening, eligibility, included in systematic review, and, if applicable, included in the meta-analysis). | 3 |
| Data collection process | 10 | Describe method of data extraction from reports (e.g., piloted forms, independently, in duplicate) and any processes for obtaining and confirming data from investigators. | 3 |
| Data items | 11 | List and define all variables for which data were sought (e.g., PICOS, funding sources) and any assumptions and simplifications made. | 3 |
| Risk of bias in individual studies | 12 | Describe methods used for assessing risk of bias of individual studies (including specification of whether this was done at the study or outcome level), and how this information is to be used in any data synthesis. | 3 |
| Summary measures | 13 | State the principal summary measures (e.g., risk ratio, difference in means). | 3 |
| Synthesis of results | 14 | Describe the methods of handling data and combining results of studies, if done, including measures of consistency (e.g., I^2^) for each meta-analysis. | 3 |

**PRISMA Checklist**.

| **Section/topic** | **#** | **Checklist item** | **Reported on page #** |
| --- | --- | --- | --- |
| Risk of bias across studies | 15 | Specify any assessment of risk of bias that may affect the cumulative evidence (e.g., publication bias, selective reporting within studies). | 3 |
| Additional analyses | 16 | Describe methods of additional analyses (e.g., sensitivity or subgroup analyses, meta-regression), if done, indicating which were pre-specified. | / |
| **RESULTS** | | |  |
| Study selection | 17 | Give numbers of studies screened, assessed for eligibility, and included in the review, with reasons for exclusions at each stage, ideally with a flow diagram. | 4 |
| Study characteristics | 18 | For each study, present characteristics for which data were extracted (e.g., study size, PICOS, follow-up period) and provide the citations. | 4 |
| Risk of bias within studies | 19 | Present data on risk of bias of each study and, if available, any outcome level assessment (see item 12). | Sup Files |
| Results of individual studies | 20 | For all outcomes considered (benefits or harms), present, for each study: (a) simple summary data for each intervention group (b) effect estimates and confidence intervals, ideally with a forest plot. | Table 4 |
| Synthesis of results | 21 | Present results of each meta-analysis done, including confidence intervals and measures of consistency. | / |
| Risk of bias across studies | 22 | Present results of any assessment of risk of bias across studies (see Item 15). | / |
| Additional analysis | 23 | Give results of additional analyses, if done (e.g., sensitivity or subgroup analyses, meta-regression [see Item 16]). | / |
| **DISCUSSION** | | |  |
| Summary of evidence | 24 | Summarize the main findings including the strength of evidence for each main outcome; consider their relevance to key groups (e.g., healthcare providers, users, and policy makers). | 18 |
| Limitations | 25 | Discuss limitations at study and outcome level (e.g., risk of bias), and at review-level (e.g., incomplete retrieval of identified research, reporting bias). | 18, 20-21 |
| Conclusions | 26 | Provide a general interpretation of the results in the context of other evidence, and implications for future research. | 21 |
| **FUNDING** | | |  |
| Funding | 27 | Describe sources of funding for the systematic review and other support (e.g., supply of data); role of funders for the systematic review. | 22 |

*From:*  Moher D, Liberati A, Tetzlaff J, Altman DG, The PRISMA Group (2009). Preferred Reporting Items for Systematic Reviews and Meta-Analyses: The PRISMA Statement. PLoS Med 6(7): e1000097. doi:10.1371/journal.pmed1000097

For more information, visit: **www.prisma-statement.org**.

### Quality assessment method by Kmet et al. (2004)

The articles’ quality was assessed with the quality assessment criteria developed by Kmet et al. (2004).

- Is the objective of the study sufficiently described?
- Is the study design evident and appropriate?
- Is the method of subject selection described and appropriate?
- If interventional and random allocation was possible, was it described?
- If interventional and blinding of investigators was possible, was it reported?
- If interventional and blinding of subjects was possible, was it reported?
- Are subject characteristics sufficiently described?
- Are outcome measures well defined and robust to measurement?
- Is the sample size appropriate?
- Are analytic methods described, justified and appropriate?
- Is some estimate of variance reported for main results?
- Are they controlled for confounding?
- Are the results reported in sufficient detail?
- Are the conclusions supported by results?

Each question can be answered with “yes”, “partial”, “no” and “not applicable”.

The associated scoring manual of Kmet et al. (2004) was used to calculate the quality score as described below:

$$Quality score=\frac{2*\left( number of ‘yes’ \right)+(number of ‘partial’)}{28-2*(number of ‘not applicable’ )}$$

In addition, the description quality of the DIY fortification recipes (fortificants, food matrices, concentration) was assessed (but not included in the quality score).

Kmet LM, Lee RC, Cook LS, Alberta Heritage Foundation for Medical Research A, Health Technology Assessment Unit, University of Calgary, et al. Standard quality assessment criteria for evaluating primary research papers from a variety of fields. Edmonton: Alberta Heritage Foundation for Medical Research; 2004.

### Quality assessment for articles related to nutritional outcomes.

| **Author(s) (year)** | ***Fortification description*** | **Objective sufficiently described?** | **Study design evident and appropriate?** | **Recruitment described and appropriate?** | **Subjects are sufficiently described?** | **If randomization possible was it done and described?** | **If possible, investigator's blinding** | **If possible, subjects' blinding** | **Outcome measures(s) well defined and robust?** | **Sample size appropriate?** | **Analytic methods described and appropriate?** | **Some estimate of variance is reported for main results?** | **Controlled for confounding?** | **Results reported in sufficient detail?** | **Conclusions supported by results?** | **Sum score** |
| --- | --- | --- | --- | --- | --- | --- | --- | --- | --- | --- | --- | --- | --- | --- | --- | --- |
| Allepaerts et al. (2020) | ***N*** | Y | Y | P | Y | NA | NA | NA | Y | P | Y | Y | Y | Y | Y | **1.00** |
| Arjuna et al. (2018) | ***P*** | Y | Y | Y | Y | P | Y | Y | Y | Y | Y | Y | Y | Y | Y | **1.00** |
| Barton et al. (2000) | ***P*** | Y | P | N | N | P | Y | Y | Y | P | Y | Y | N | Y | Y | **0.79** |
| Beelen et al. (2017a) | ***Y*** | Y | P | Y | Y | NA | NA | NA | Y | Y | Y | Y | Y | Y | Y | **1.00** |
| Beelen et al. (2017b) | ***N*** | Y | Y | P | Y | Y | N | NA | Y | Y | Y | Y | Y | Y | Y | **0.92** |
| Beelen et al. (2018) | ***N*** | Y | Y | P | Y | Y | N | NA | Y | Y | Y | Y | P | Y | Y | **0.92** |
| Beerman et al. (2016) | ***P*** | Y | P | P | P | NA | NA | NA | Y | Y | Y | Y | Y | Y | Y | **1.00** |
| Björkman et al. (2012) | ***Y*** | Y | Y | P | Y | Y | Y | Y | Y | Y | Y | Y | Y | Y | Y | **1.00** |
| Bonnefoy et al. (2010) | ***Y*** | Y | Y | N | Y | P | N | NA | Y | Y | Y | Y | Y | Y | Y | **0.85** |
| Castellanos et al. (2009) | ***P*** | Y | Y | N | P | P | NA | Y | Y | N | Y | Y | Y | Y | Y | **0.85** |
| Evans et al. (2017) | ***Y*** | Y | Y | P | Y | Y | Y | Y | Y | P | Y | Y | Y | Y | Y | **1.00** |
| Gall et al. (1998) | ***P*** | Y | P | Y | P | N | N | NA | Y | Y | Y | Y | P | Y | Y | **0.85** |
| Hashimoto et al. (2015) | ***P*** | Y | N | P | Y | N | N | NA | Y | N | Y | Y | P | Y | Y | **0.69** |
| Irvine et al. (2004) | ***P*** | Y | N | N | P | Y | N | NA | Y | N | Y | Y | N | Y | Y | **0.62** |
| Iuliano et al. (2013) | ***P*** | Y | Y | P | Y | P | N | NA | Y | Y | P | Y | P | P | Y | **0.92** |
| Lee et al. (2013) | ***P*** | Y | Y | P | Y | P | Y | Y | Y | Y | Y | Y | Y | Y | Y | **1.00** |
| Leslie et al. (2013) | ***Y*** | Y | Y | Y | P | Y | N | NA | Y | Y | Y | Y | P | Y | Y | **0.92** |
| Lorefält et al. (2005) | ***P*** | Y | Y | N | Y | NA | NA | NA | Y | N | Y | Y | Y | Y | Y | **0.82** |
| Mertz et al. (2021) | ***P*** | Y | Y | Y | Y | Y | Y | Y | Y | Y | Y | Y | N | Y | Y | **0.93** |
| Mortensen et al. (2019) | ***P*** | Y | Y | P | Y | NA | NA | NA | Y | Y | Y | Y | Y | Y | Y | **1.00** |
| Munk et al. (2013) | ***N*** | Y | P | Y | Y | NA | N | NA | Y | Y | Y | NA | Y | P | Y | **0.91** |
| Munk et al. (2014) | ***P*** | Y | Y | P | Y | Y | P | NA | Y | Y | Y | Y | Y | Y | Y | **1.00** |
| Neelemaat et al. (2012) | ***P*** | Y | Y | P | Y | Y | N | NA | Y | Y | Y | Y | Y | Y | Y | **0.92** |
| Niccoli et al. (2017) | ***Y*** | Y | Y | P | Y | Y | Y | Y | Y | P | Y | Y | Y | Y | Y | **1.00** |
| Nykänen et al. (2019) | ***P*** | Y | Y | P | Y | Y | N | NA | Y | Y | Y | Y | Y | Y | Y | **0.92** |
| Ödlund Olin (2003) | ***Y*** | Y | Y | N | Y | NA | N | NA | Y | P | Y | Y | P | Y | Y | **0.83** |
| Ödlund Olin et al. (1996) | ***Y*** | Y | Y | N | Y | N | P | P | Y | P | Y | Y | Y | Y | Y | **0.86** |
| Ott et al. (2019) | ***P*** | Y | Y | P | Y | NA | NA | NA | Y | N | Y | Y | N | Y | Y | **0.82** |
| Park et al. (2018) | ***Y*** | Y | Y | P | Y | Y | Y | Y | Y | Y | Y | Y | P | Y | Y | **1.00** |
| Polonen et al. (2017) | ***P*** | Y | Y | N | Y | NA | N | NA | Y | Y | Y | Y | Y | Y | Y | **0.83** |
| Seemer et al. (2021) | ***Y*** | Y | Y | P | Y | NA | NA | NA | Y | Y | Y | Y | Y | Y | Y | **1.00** |
| Silver et al. (2008) | ***Y*** | Y | Y | P | P | Y | NA | NA | Y | Y | Y | Y | P | Y | Y | **1.00** |
| Smoliner et al. (2008) | ***Y*** | Y | P | N | Y | Y | N | NA | Y | Y | Y | Y | Y | Y | Y | **0.85** |
| Sossen et al. (2020) | ***P*** | Y | Y | P | Y | NA | N | NA | Y | Y | Y | Y | Y | Y | Y | **0.92** |
| Starke et al. (2011) | ***P*** | Y | Y | P | P | Y | P | NA | Y | Y | Y | Y | Y | Y | Y | **1.00** |
| Stelten et al. (2015) | ***Y*** | Y | Y | Y | Y | Y | N | Y | Y | P | Y | Y | Y | Y | Y | **0.93** |
| Stow et al. (2015) | ***Y*** | Y | Y | Y | P | Y | N | Y | Y | Y | P | Y | P | Y | Y | **0.93** |
| Van Til et al. (2015) | ***Y*** | Y | Y | Y | Y | Y | N | Y | Y | P | Y | Y | Y | Y | Y | **0.93** |
| Young et al. (2018) | ***N*** | Y | P | P | Y | NA | N | NA | Y | Y | Y | Y | Y | Y | Y | **0.92** |
| Ziylan et al. (2016) | ***P*** | Y | Y | Y | Y | P | NA | Y | Y | Y | Y | Y | Y | Y | Y | **1.00** |
| Ziylan et al. (2017) | ***P*** | Y | Y | P | Y | Y | Y | Y | Y | P | Y | Y | Y | Y | Y | **1.00** |

Evaluation of the quality of the 14 items of the Standard Quality Assessment Criteria (Kmet et al, 2004); good quality (Y), medium quality (P), bad quality (N). Sum score close to 1 corresponds to a good quality and close to 0 to a bad quality. The quality variable “fortification description” was not used to calculate the sum score.

### Quality assessment for articles related to acceptability outcome.

| **Author(s) (year)** | ***Fortification description*** | **Objective sufficiently described?** | **Study design evident and appropriate?** | **Recruitment described and appropriate?** | **Subjects are sufficiently described?** | **If randomization possible was it done and described?** | **If possible, investigator's blinding** | **If possible, subjects' blinding** | **Outcome measures(s) well defined and robust?** | **Sample size appropriate?** | **Analytic methods described and appropriate?** | **Some estimate of variance is reported for main results?** | **Controlled for confounding?** | **Results reported in sufficient detail?** | **Conclusions supported by results?** | **Sum score** |
| --- | --- | --- | --- | --- | --- | --- | --- | --- | --- | --- | --- | --- | --- | --- | --- | --- |
| Arjuna et al. (2018) | ***P*** | Y | P | Y | Y | NA | NA | NA | P | N | P | N | NA | P | Y | **0.80** |
| Beelen et al. (2017a) | ***Y*** | Y | N | Y | Y | NA | NA | NA | N | Y | P | NA | NA | N | Y | **0.67** |
| Castellanos et al. (2009) | ***P*** | Y | Y | N | N | N | NA | N | Y | N | N | N | NA | P | Y | **0.42** |
| Munk et al. (2013) | ***N*** | Y | Y | P | N | N | NA | N | Y | Y | N | N | NA | N | Y | **0.50** |
| Norton et al. (2020) | ***P*** | Y | Y | P | Y | Y | NA | Y | Y | Y | Y | Y | Y | Y | Y | **1.00** |
|  | ***P*** | Y | Y | P | Y | Y | NA | Y | Y | Y | Y | Y | Y | Y | Y | **1.00** |
| Nykänen et al. (2019) | ***P*** | Y | P | P | Y | NA | NA | NA | P | P | N | N | NA | P | Y | **0.80** |
| Ott et al. (2019) | ***P*** | Y | N | N | N | NA | NA | NA | N | P | P | NA | NA | P | Y | **0.56** |
| Silver et al. (2008) | ***Y*** | Y | N | P | N | N | NA | N | P | N | N | N | NA | N | Y | **0.33** |
| Stelten et al. (2015) | ***Y*** | Y | P | Y | Y | NA | NA | NA | P | N | Y | N | NA | P | Y | **0.80** |
| Tsikritzi et al. (2015) | ***Y*** | Y | Y | N | P | Y | NA | Y | Y | P | Y | N | NA | P | Y | **0.83** |
| Wendin et al. (2017) | ***Y*** | Y | P | N | P | NA | NA | P | P | Y | N | NA | NA | Y | Y | **0.80** |
| Ziylan et al. (2016) | ***P*** | Y | Y | Y | Y | P | NA | Y | Y | Y | Y | Y | Y | Y | Y | **1.00** |
| Ziylan et al. (2017) | ***P*** | Y | Y | P | Y | NA | NA | NA | Y | P | Y | Y | Y | Y | Y | **1.00** |

Evaluation of the quality of the 14 items of the Standard Quality Assessment Criteria (Kmet et al, 2004); good quality (Y), medium quality (P), bad quality (N). Sum score close to 1 corresponds to a good quality and close to 0 to a bad quality. The quality variable “fortification description” was not used to calculate the sum score.

### Articles excluded after full-text screening and reasons of exclusions

| **Article (full reference)** | **Reasons of exclusion** |
| --- | --- |
| [Goineau, C. (2010). Can an increase in the energy content of food present an alternative to oral food supplements? [L’augmentation de la densité énergétique des aliments peut-elle être une alternative aux compléments nutritionnels oraux?]. Cahiers de l’Annee Gerontologique, 2(3), 217–219. https://doi.org/10.1007/s12612-010-0164-z](https://doi.org/10.1007/s12612-010-0164-z) | Foreign language |
| [Lecerf, J.-M., Dalle, B., Padol, J., Berthier, C., Jaruga, A., Jozeau, P., Herault, S., & Deparis, F. (2017). An evening meal formula against poor nutrition in nursing homes [Une formule de repas du soir contre la sous-nutrition en EHPAD]. Nutrition Clinique et Metabolisme, 31(3), 212–217. https://doi.org/10.1016/j.nupar.2017.06.090](https://doi.org/10.1016/j.nupar.2017.06.090) | Foreign language |
| [Lefort, A. (2010). Effects of whey and fortified collagen hydrolysate protein supplements on nitrogen balance and body composition in elderly women [Effets du lactosérum et des compléments protéiques au collagène fortifié sur la balance azotée et la composition corporelle chez les femmes âgées]. Cahiers de l’Annee Gerontologique, 2(3), 205–207. https://doi.org/10.1007/s12612-010-0064-2](https://doi.org/10.1007/s12612-010-0064-2) | Foreign language |
| [Prêcheur, I., Brocker, P., Schneider, S. M., Barthélémi, C., Bertoglio, J., Philip, J.-L., Solere, J.-P., Manckoundia, P., Van Wymelbeke, V., Amar, P., Darque-Ceretti, E., & Pesci-Bardon, C. (2014). A solid oral nutritional supplement to increase protein and energy intakes independently of dental health status [Un complément nutritionnel oral solide pour renforcer l’apport protéino-énergétique quel que soit l’état dentaire]. Cahiers de Nutrition et de Dietetique, 49(3), 130–138. https://doi.org/10.1016/j.cnd.2014.01.003](https://doi.org/10.1016/j.cnd.2014.01.003) | Foreign language |
| [Raimbault, F., Schonheit, C., Curtis, V., Sczepanek, D., Loustau, M., & Mézière, A. (2017). Dietary enrichment after osteoporotic fracture during hospitalization in orthogeriatric rehabilitation care unit: Nutritional and functional interest? [Enrichissement alimentaire après fracture ostéoporotique au cours d’une réhabilitation en soins de suite et réadaptation orthogériatrique: Intérêt nutritionnel et/ou fonctionnel ?]. Nutrition Clinique et Metabolisme, 31(2), 151–161. https://doi.org/10.1016/j.nupar.2017.02.001](https://doi.org/10.1016/j.nupar.2017.02.001) | Foreign language |
| Schürch, M.-A., Bonjour, J.-P., Slosman, D., & Rizzoli, R. (1999). Protein supplements favorably influence the outcome after hip fracture [Un supplement proteique ameliore les suites d’une fracture du femur proximal]. Medecine et Hygiene, 57(2271), 1876–1878. | Foreign language |
| Van Wymelbeke, V., Lesourd, B., Bon, F., & Martin-Pfitzenmeyer, I. (2013). Elderly population and health professionals: The difference of taste [Population âgée et professionnels de santé: La différence de goût]. Revue de Geriatrie, 38(10), 769–772. | Foreign language |
| [Chin A Paw, M. J. M., De Jong, N., Schouten, E. G., Hiddink, G. J., & Kok, F. J. (2001). Physical exercise and/or enriched foods for functional improvement in frail, independently living elderly: A randomized controlled trial. Archives of Physical Medicine and Rehabilitation, 82(6), 811–817. https://doi.org/10.1053/apmr.2001.23278](https://doi.org/10.1053/apmr.2001.23278) | Micronutrients solely |
| [De jong, N. (2001). Sensible aging: Using nutrient-dense foods and physical exercise with the frail elderly. Nutrition Today, 36(4), 105A. https://doi.org/10.1097/00017285-200107000-00004](https://doi.org/10.1097/00017285-200107000-00004) | Micronutrients solely |
| [de Jong, N., Chin A Paw, M. J., de Graaf, C., & van Staveren, W. A. (2000). Effect of dietary supplements and physical exercise on sensory perception, appetite, dietary intake and body weight in frail elderly subjects. The British Journal of Nutrition, 83(6), 605–613. https://doi.org/10.1017/s0007114500000775](https://doi.org/10.1017/s0007114500000775) | Micronutrients solely |
| [de Jong, N., Chin A Paw, M. J., de Graaf, C., Hiddink, G. J., de Groot, L., & van Staveren, W. A. (2001). Appraisal of 4 months’ consumption of nutrient-dense foods within the daily feeding pattern of frail elderly. Journal of Aging and Health, 13(2), 200–216. https://doi.org/10.1177/089826430101300203](https://doi.org/10.1177/089826430101300203) | Micronutrients solely |
| [de Jong, N., Chin A Paw, M. J., de Groot, L. C., de Graaf, C., Kok, F. J., & van Staveren, W. A. (1999). Functional biochemical and nutrient indices in frail elderly people are partly affected by dietary supplements but not by exercise. The Journal of Nutrition, 129(11), 2028–2036. https://doi.org/10.1093/jn/129.11.2028](https://doi.org/10.1093/jn/129.11.2028) | Micronutrients solely |
| [de Jong, N., Chin A Paw, M. J., de Groot, L. C., Hiddink, G. J., & van Staveren, W. A. (2000). Dietary supplements and physical exercise affecting bone and body composition in frail elderly persons. American Journal of Public Health, 90(6), 947–954. https://doi.org/10.2105/ajph.90.6.947](https://doi.org/10.2105/ajph.90.6.947) | Micronutrients solely |
| Katakity, M., Webb, J. F., & Dickerson, J. W. T. (1983). Some effects of a food supplement in elderly hospital patients: A longitudinal study. Human Nutrition: Applied Nutrition, 37(2), 85–93. | Micronutrients solely |
| [Kukuljan, S., Nowson, C. A., Sanders, K., & Daly, R. M. (2009). Effects of resistance exercise and fortified milk on skeletal muscle mass, muscle size, and functional performance in middle-aged and older men: An 18-mo randomized controlled trial. Journal of Applied Physiology (Bethesda, Md. : 1985), 107(6), 1864–1873. https://doi.org/10.1152/japplphysiol.00392.2009](https://doi.org/10.1152/japplphysiol.00392.2009) | Micronutrients solely |
| Kwok, T., Woo, J., & Kwan, M. (2001). Does low lactose milk powder improve the nutritional intake and nutritional status of frail older Chinese people living in nursing homes? Journal of Nutrition, Health and Aging, 5(1), 17–21. | Micronutrients solely |
| [Manders, M., de Groot, C. P. G. M., Blauw, Y. H., Dhonukshe-Rutten, R. A. M., van Hoeckel-Prüst, L., Bindels, J. G., Siebelink, E., & van Staveren, W. A. (2009). Effect of a nutrient-enriched drink on dietary intake and nutritional status in institutionalised elderly. European Journal of Clinical Nutrition, 63(10), 1241–1250. https://doi.org/10.1038/ejcn.2009.28](https://doi.org/10.1038/ejcn.2009.28) | Micronutrients solely |
| [Manders, M., De Groot, L. C. P. G. M., Hoefnagels, W. H. L., Dhonukshe-Rutten, R. A. M., Wouters-Wesseling, W., Mulders, A. J. M. J., & Van Staveren, W. A. (2009). The effect of a nutrient dense drink on mental and physical function in institutionalized elderly people. The Journal of Nutrition, Health & Aging, 13(9), 760–767. https://doi.org/10.1007/s12603-009-0211-x](https://doi.org/10.1007/s12603-009-0211-x) | Micronutrients solely |
| Nutrition in Disguise: Effects of Food Neophobia, Healthy Eating Interests and Provision of Health Information on Liking and Perceptions of Nutrient-Dense Foods in Older Adults | Micronutrients solely |
| [Romaniw, O. C., Rajpal, R., Duncan, A. M., Keller, H. H., & Duizer, L. M. (2020). Nutrition in Disguise: Effects of Food Neophobia, Healthy Eating Interests and Provision of Health Information on Liking and Perceptions of Nutrient-Dense Foods in Older Adults. Foods (Basel, Switzerland), 10(1). https://doi.org/10.3390/foods10010060](https://doi.org/10.3390/foods10010060) | Micronutrients solely |
| van der Wielen, R. P., van Heereveld, H. A., de Groot, C. P., & van Staveren, W. A. (1995). Nutritional status of elderly female nursing home residents; the effect of supplementation with a physiological dose of water-soluble vitamins. European Journal of Clinical Nutrition, 49(9), 665–674. | Micronutrients solely |
| [Whiting, S. J., Kohrt, W. M., Warren, M. P., Kraenzlin, M. I., & Bonjour, J.-P. (2016). Food fortification for bone health in adulthood: A scoping review. European Journal of Clinical Nutrition, 70(10), 1099–1105. https://doi.org/10.1038/ejcn.2016.42](https://doi.org/10.1038/ejcn.2016.42) | Micronutrients solely |
| A Novel Fortified Dairy Product and Sarcopenia Measures in Sarcopenic Older Adults: A Double-Blind Randomized Controlled Trial | No fortification |
| [Alemán-Mateo, H., Carreón, V. R., Macías, L., Astiazaran-García, H., Gallegos-Aguilar, A. C., & Enríquez, J. R. R. (2014). Nutrient-rich dairy proteins improve appendicular skeletal muscle mass and physical performance, And attenuate the loss of muscle strength in older men and women subjects: A single-blind randomized clinical trial. Clinical Interventions in Aging, 9, 1517–1525. https://doi.org/10.2147/CIA.S67449](https://doi.org/10.2147/CIA.S67449) | No fortification |
| [Alemán-Mateo, H., Macías, L., Esparza-Romero, J., Astiazaran-García, H., & Blancas, A. L. (2012). Physiological effects beyond the significant gain in muscle mass in sarcopenic elderly men: Evidence from a randomized clinical trial using a protein-rich food. Clinical Interventions in Aging, 7, 225–234. https://doi.org/10.2147/CIA.S32356](https://doi.org/10.2147/CIA.S32356) | No fortification |
| Appleton KM. 2009. Increases in energy, protein and fat intake following the addition of sauce to an older person’s meal. Appetite 52:161–5. | No fortification |
| [Bae, M. A., Park, S. H., Han, S. H., Chang, K. J., & Kim, S. H. (2019). Food Preference of the Elderly for the Development of Taurine-Containing Elderly-Friendly Foods. Advances in Experimental Medicine and Biology, 1155, 249–259. https://doi.org/10.1007/978-981-13-8023-5_24](https://doi.org/10.1007/978-981-13-8023-5_24) | No fortification |
| Bakhtiari A, Yassin Z, Hanachi P, et al. (2012) Effects of soy on body composition: a 12-week randomized controlled trial among Iranian elderly women with metabolic syndrome. | No fortification |
| [Banovic, M., Arvola, A., Pennanen, K., Duta, D. E., Brückner-Gühmann, M., Lähteenmäki, L., & Grunert, K. G. (2018). Foods with increased protein content: A qualitative study on European consumer preferences and perceptions. Appetite, 125, 233–243. https://doi.org/10.1016/j.appet.2018.01.034](https://doi.org/10.1016/j.appet.2018.01.034) | No fortification |
| [Barichella, M., Cereda, E., Pinelli, G., Iorio, L., Caroli, D., Masiero, I., Ferri, V., Cassani, E., Bolliri, C., Caronni, S., Maggio, M., Ortelli, P., Ferrazzoli, D., Maras, A., Riboldazzi, G., Frazzitta, G., & Pezzoli, G. (2019). Muscle-targeted nutritional support for rehabilitation in patients with parkinsonian syndrome. Neurology, 93(5), e485–e496. https://doi.org/10.1212/WNL.0000000000007858](https://doi.org/10.1212/WNL.0000000000007858) | No fortification |
| [Beck, A. M., Ovesen, L., & Schroll, M. (2002). Home-made oral supplement as nutritional support of old nursing home residents, who are undernourished or at risk of undernutrition based on the MNA. A pilot trial. Aging Clinical and Experimental Research, 14(3), 212–215. https://doi.org/10.1007/BF03324439](https://doi.org/10.1007/BF03324439) | No fortification |
| Beck, A.M.; Christensen, A.G.; Hansen, B.S.; Damsbo-Svendsen, S.; Moller, T.K. Multidisciplinary nutritional support for undernutrition in nursing home and home-care: A cluster randomized controlled trial. Nutrition 2016, 32, 199–205. | No fortification |
| Beck, A.M.; Damkjaer, K.; Sorbye, L.W. Physical and social functional abilities seem to be maintained by a multifaceted randomized controlled nutritional intervention among old (>65 years) Danish nursing home residents. Arch. Gerontol. Geriatr. 2010, 50, 351–355. | No fortification |
| Bell KE, Snijders T, Zulyniak M, Kumbhare D, Parise G, Chabowski A, Phillips SM. A whey protein-based multi-ingredient nutritional supplement stimulates gains in lean body mass and strength in healthy older men: a randomized controlled trial. PLoS One 2017;12:e0181387. | No fortification |
| [Beriain, Ma. J., Ibanez, F. C., Baleztena, J., & Oria, E. (2011). The effect of a modified meat product on nutritional status in institutionalized elderly people. In Nutricion Hospitalaria (Vol. 26, Issue 4, pp. 907–915). ARAN EDICIONES, S L. https://doi.org/10.3305/nh.2011.26.4.5142](https://doi.org/10.3305/nh.2011.26.4.5142) | No fortification |
| Best RL, Appleton KM. 2011. Comparable increases in energy, protein and fat intakes following the addition of seasonings and sauces to an older person’s meal. Appetite 56: 179–82. | No fortification |
| [Bitok, E., Rajaram, S., Jaceldo-Siegl, K., Oda, K., Sala-Vila, A., Serra-Mir, M., Ros, E., & Sabaté, J. (2018). Effects of long-term walnut supplementation on body weight in free-living elderly: Results of a randomized controlled trial. Nutrients, 10(9). https://doi.org/10.3390/nu10091317](https://doi.org/10.3390/nu10091317) | No fortification |
| [Björkman, M. P., Pilvi, T. K., Kekkonen, R. A., Korpela, R., & Tilvis, R. S. (2011). Similar effects of leucine rich and regular dairy products on muscle mass and functions of older polymyalgia rheumatica patients: A randomized crossover trial. The Journal of Nutrition, Health & Aging, 15(6), 462–467. https://doi.org/10.1007/s12603-010-0276-6](https://doi.org/10.1007/s12603-010-0276-6) | No fortification |
| [Bos, C., Benamouzig, R., Bruhat, A., Roux, C., Mahé, S., Valensi, P., Gaudichon, C., Ferrière, F., Rautureau, J., & Tomé, D. (2000). Short-term protein and energy supplementation activates nitrogen kinetics and accretion in poorly nourished elderly subjects. American Journal of Clinical Nutrition, 71(5), 1129–1137. https://doi.org/10.1093/ajcn/71.5.1129](https://doi.org/10.1093/ajcn/71.5.1129) | No fortification |
| [Buckinx, F., Carvalho, L. P., Marcangeli, V., Dulac, M., G., H. B., Gouspillou, G., Gaudreau, P., Noirez, P., & M., A.-L. (2020). High intensity interval training combined with L-citrulline supplementation: Effects on physical performance in healthy older adults. Experimental Gerontology, 140. https://doi.org/10.1016/j.exger.2020.111036](https://doi.org/10.1016/j.exger.2020.111036) | No fortification |
| Bukhari, S.S.I.; Phillips, B.E.; Wilkinson, D.J.; Limb, M.C.; Rankin, D.; Mitchell, W.K.; Kobayashi, H.; Greenha, P.L.; Smith, K.; Atherton, P.J. Intake of low-dose leucine-rich essential amino acids stimulates muscle anabolism equivalently to bolus whey protein in older women at rest and after exercise. Am. J. Physiol. Endocrinol. Metab. 2015, 308, E1056–E1065. | No fortification |
| [Buondonno, I., Sassi, F., Carignano, G., Dutto, F., Ferreri, C., Pili, F. G., Massaia, M., Nisoli, E., Ruocco, C., Porrino, P., Ravetta, C., Riganti, C., Isaia, G. C., & D’Amelio, P. (2020). From mitochondria to healthy aging: The role of branched-chain amino acids treatment: MATeR a randomized study. Clinical Nutrition, 39(7), 2080–2091. https://doi.org/10.1016/j.clnu.2019.10.013](https://doi.org/10.1016/j.clnu.2019.10.013) | No fortification |
| Campbell WW, Crim MC, Young VR, Joseph LJ, Evans WJ. Effects of resistance training and dietary protein intake on protein metabolism in older adults. Am J Physiol 1995;268: 1143-1153. PMID: 7611390 | No fortification |
| Candow DG, Chilibeck PD, Facci M, Abeysekara S, Zello GA. Protein supplementation before and after resistance training in older men. Eur J Appl Physiol 2006;97: 548-556. doi: 10.1007/s00421-006-0223-8 | No fortification |
| [Carlsson, M., Littbrand, H., Gustafson, Y., Lundin-Olsson, L., Lindelöf, N., Rosendahl, E., & Håglin, L. (2011). Effects of high-intensity exercise and protein supplement on muscle mass in ADL dependent older people with and without malnutrition: A randomized controlled trial. The Journal of Nutrition, Health & Aging, 15(7), 554–560. https://doi.org/10.1007/s12603-011-0017-5](https://doi.org/10.1007/s12603-011-0017-5) | No fortification |
| Chanet A, Verlaan S, Salles J, Giraudet C, Patrac V, Pidou V, Pouyet C, Hafnaoui N, Blot A, Cano N, et al. Supplementing breakfast with a vitamin D and leucine-enriched whey protein medical nutrition drink enhances postprandial muscle protein synthesis and muscle mass in healthy older men. J Nutr 2017;147:2262–7 | No fortification |
| [Charlton, K. E., Walton, K., Moon, L., Smith, K., McMahon, A. T., Ralph, F., Stuckey, M., Manning, F., & Krassie, J. (2013). “It could probably help someone else but not me”: A feasibility study of a snack programme offered to meals on wheels clients. Journal of Nutrition, Health and Aging, 17(4), 364–369. https://doi.org/10.1007/s12603-013-0035-6](https://doi.org/10.1007/s12603-013-0035-6) | No fortification |
| Corcoran, M.P.; Nelson, M.E.; Sacheck, J.M.; Reid, K.F.; Kirn, D.; Fielding, R.A.; Chui, K.K.H.; Folta, S.C. Efficacy of an Exercise and Nutritional Supplement Program on Physical Performance and Nutritional Status in Older AdultsWith Mobility Limitations Residing at Senior Living Facilities. J. Aging Phys. Act. 2017, 25, 453–463. | No fortification |
| [Daly, R. M., O’Connell, S. L., Mundell, N. L., Grimes, C. A., Dunstan, D. W., & Nowson, C. A. (2014). Protein-enriched diet, with the use of lean red meat, combined with progressive resistance training enhances lean tissue mass and muscle strength and reduces circulating IL-6 concentrations in elderly women: A cluster randomized controlled trial. In American Journal of Clinical Nutrition (Vol. 99, Issue 4, pp. 899–910). OXFORD UNIV PRESS. https://doi.org/10.3945/ajcn.113.064154](https://doi.org/10.3945/ajcn.113.064154) | No fortification |
| [Dillon, E. L., Sheffield-Moore, M., Paddon-Jones, D., Gilkison, C., Sanford, A. P., Casperson, S. L., Jiang, J., Chinkes, D. L., & Urban, R. J. (2009). Amino acid supplementation increases lean body mass, basal muscle protein synthesis, and insulin-like growth factor-I expression in older women. Journal of Clinical Endocrinology and Metabolism, 94(5), 1630–1637. https://doi.org/10.1210/jc.2008-1564](https://doi.org/10.1210/jc.2008-1564) | No fortification |
| Dirks, M.L.; Tieland, M.; Verdijk, L.B.; Losen, M.; Nilwik, R.; Mensink, M.; de Groot, L.C.P.G.M.; van Loon, L.J.C. Protein Supplementation Augments Muscle Fiber Hypertrophy but Does Not Modulate Satellite Cell Content During Prolonged Resistance-Type Exercise Training in Frail Elderly. J. Am. Med. Dir. Assoc. 2017, 18, 608–615. | No fortification |
| [Donahue, E., Crowe, K. M., & Lawrence, J. (2015). Protein-enhanced soups: A consumer-accepted food for increasing dietary protein provision among older adults. In International Journal of Food Sciences and Nutrition (Vol. 66, Issue 1, pp. 104–107). TAYLOR & FRANCIS LTD. https://doi.org/10.3109/09637486.2014.953451](https://doi.org/10.3109/09637486.2014.953451) | No fortification |
| [Doorduijn, A. S., van Gameren, Y., Vasse, E., & de Roos, N. M. (2016). At Your Request(®) room service dining improves patient satisfaction, maintains nutritional status, and offers opportunities to improve intake. Clinical Nutrition (Edinburgh, Scotland), 35(5), 1174–1180. https://doi.org/10.1016/j.clnu.2015.10.009](https://doi.org/10.1016/j.clnu.2015.10.009) | No fortification |
| [Edington, J., Barnes, R., Bryan, F., Dupree, E., Frost, G., Hickson, M., Lancaster, J., Mongia, S., Smith, J., Torrance, A., West, R., Pang, F., & Coles, S. J. (2004). A prospective randomised controlled trial of nutritional supplementation in malnourished elderly in the community: Clinical and health economic outcomes. Clinical Nutrition (Edinburgh, Scotland), 23(2), 195–204. https://doi.org/10.1016/S0261-5614(03)00107-9](https://doi.org/10.1016/S0261-5614(03)00107-9) | No fortification |
| Effects of low-dose milk protein supplementation following low-to-moderate intensity exercise training on muscle mass in healthy older adults: a randomized placebo-controlled trial | No fortification |
| Elmstahl S, Steen B: Hospital nutrition in geriatric long-term care medicine. Effects of dietary supplements. Age Ageing 16:73-80, 1987 | No fortification |
| Espaulella J, Guyer H, Diaz-Escriu F, et al. (2000) Nutritional supplementation of elderly hip fracture patients. A randomized, double-blind, placebo-controlled trial | No fortification |
| [Fabian, E., Gerstorfer, I., Thaler, H. W., Stundner, H., Biswas, P., & Elmadfa, I. (2011). Nutritional supplementation affects postoperative oxidative stress and duration of hospitalization in patients with hip fracture. Wiener Klinische Wochenschrift, 123(3–4), 88–93. https://doi.org/10.1007/s00508-010-1519-6](https://doi.org/10.1007/s00508-010-1519-6) | No fortification |
| [Farouk, M. M., Yoo, M. J. Y., Hamid, N. S. A., Staincliffe, M., Davies, B., & Knowles, S. O. (2018). Novel meat-enriched foods for older consumers. Food Research International, 104, 134–142. https://doi.org/10.1016/j.foodres.2017.10.033](https://doi.org/10.1016/j.foodres.2017.10.033) | No fortification |
| [Fiatarone, M. A., O’Neill, E. F., Doyle, N., Clements, K. M., Roberts, S. B., Kehayias, J. J., Lipsitz, L. A., & Evans, W. J. (1993). The Boston FICSIT study: The effects of resistance training and nutritional supplementation on physical frailty in the oldest old. Journal of the American Geriatrics Society, 41(3), 333–337. https://doi.org/10.1111/j.1532-5415.1993.tb06714.x](https://doi.org/10.1111/j.1532-5415.1993.tb06714.x) | No fortification |
| [Fiatarone, M. A., O’Neill, E. F., Ryan, N. D., Clements, K. M., Solares, G. R., Nelson, M. E., Roberts, S. B., Kehayias, J. J., Lipsitz, L. A., & Evans, W. J. (1994). Exercise training and nutritional supplementation for physical frailty in very elderly people. The New England Journal of Medicine, 330(25), 1769–1775. https://doi.org/10.1056/NEJM199406233302501](https://doi.org/10.1056/NEJM199406233302501) | No fortification |
| Gariballa S, Forster S. Dietary supplementation and quality of life of older patients: A randomized, double-blind, placebo-controlled trial. J Am Geriatr Soc. 2007;55:2030– 4. | No fortification |
| [GERICKE, O. L., LOBB, L. G., & ALLINGER, D. E. (1961). NUTRITIONAL SUPPLEMENTATION FOR ELDERLY PATIENTS IN A STATE MENTAL HOSPITAL: EFFECT ON APPETITE AND WEIGHT GAIN. Journal of the American Geriatrics Society, 9(5), 381–387. https://doi.org/10.1111/j.1532-5415.1961.tb00474.x](https://doi.org/10.1111/j.1532-5415.1961.tb00474.x) | No fortification |
| [Gryson, C., Ratel, S., Rance, M., Penando, S., Bonhomme, C., Le Ruyet, P., Duclos, M., Boirie, Y., & Walrand, S. (2014). Four-Month Course of Soluble Milk Proteins Interacts With Exercise to Improve Muscle Strength and Delay Fatigue in Elderly Participants. Journal of the American Medical Directors Association, 15(12), 958.e1-958.e9. https://doi.org/10.1016/j.jamda.2014.09.011](https://doi.org/10.1016/j.jamda.2014.09.011) | No fortification |
| [Harper, J. R., McAlpine, S., Hetherington, M. M., Bolton-Smith, C., & McMurdo, M. E. T. (2001). Preferences for different high-energy foods in elderly medical in-patients. Scottish Medical Journal, 46(6), 171–172. https://doi.org/10.1177/003693300104600606](https://doi.org/10.1177/003693300104600606) | No fortification |
| [Hays, N. P., Kim, H., Wells, A. M., Kajkenova, O., & Evans, W. J. (2009). Effects of whey and fortified collagen hydrolysate protein supplements on nitrogen balance and body composition in older women. Journal of the American Dietetic Association, 109(6), 1082–1087. https://doi.org/10.1016/j.jada.2009.03.003](https://doi.org/10.1016/j.jada.2009.03.003) | No fortification |
| Hickson M, Bulpitt C, Nunes M, Peters R, Cooke J, Nicholl C, et al. Does additional feeding support provided by health care assistants improve nutritional status and outcomes in acutely ill older in-patients? - a randomised control trial. Clin Nutr 2004;23:69e77. | No fortification |
| Hofmann, M.; Schober-Halper, B.; Oesen, S.; Franzke, B.; Tschan, H.; Bachl, N.; Strasser, E.M.; Quittan, M.; Wagner, K.H.; Wessner, B. Effects of elastic band resistance training and nutritional supplementation on muscle quality and circulating muscle growth and degradation factors of institutionalized elderly women: The Vienna Active Ageing Study (VAAS). Eur. J. Appl. Physiol. 2016, 116, 885–897. | No fortification |
| [Holst M, Beermann T, Mortensen MN, Skadhauge LB, Lindorff-Larsen K, Rasmussen HH. Multi-modal intervention improved oral intake in hospitalized patients. A one year follow-up study. Clin Nutr 2015;34(2):315e22. https://doi.org/10.1016/j.clnu.2014.05.001](https://doi.org/10.1016/j.clnu.2014.05.001) | No fortification |
| Holst, M., Mortensen, M.N., Jacobsen, B.A., Rasmussen, H.H., 2010. Efficacy of serving bedside in-between meals – An intervention study in three medical departments. e-SPEN, the European e-Journal of Clinical Nutrition and Metabolism 5, e30–e36. | No fortification |
| Holwerda, A.M.; Paulussen, K.J.M.; Overkamp, M.; Goessens, J.P.B.; Kramer, I.-F.; Wodzig, W.K.W.H.; Verdijk, L.B.; de Groot, L.C.P.G.M.; van Loon, L.J.C. Leucine co-ingestion augments the muscle protein synthetic response to the ingestion of 15 g protein following resistance exercise in older men. Am. J. Physiol. Endocrinol. Metab. 2019 | No fortification |
| Holyday M, Daniells S, Bare M, Caplan GA, Petocz P, Bolin T. Malnutrition screening and early nutrition intervention in hospitalised patients in acute aged care: A randomised controlled trial. J Nutr Health Aging. 2012;16:562–8. | No fortification |
| Ikeda, T.; Aizawa, J.; Nagasawa, H.; Gomi, I.; Kugota, H.; Nanjo, K.; Jinno, T.; Masuda, T.; Morita, S. Effects and feasibility of exercise therapy combined with branched-chain amino acid supplementation on muscle strengthening in frail and pre-frail elderly people requiring long-term care: A crossover trial. Appl. Physiol. Nutr. Metabol. 2016, 41, 438–445. | No fortification |
| Imaoka, M.; Higuchi, Y.; Todo, E.; Kitagwa, T.; Ueda, T. Low-frequency Exercise and Vitamin D Supplementation Reduce Falls Among Institutionalized Frail Elderly. Int. J. Gerontol. 2016, 10, 202–206. | No fortification |
| [Ispoglou, T., Deighton, K., King, R. F., White, H., & Lees, M. (2017). Novel essential amino acid supplements enriched with L-leucine facilitate increased protein and energy intakes in older women: A randomised controlled trial. Nutrition Journal, 16(1). https://doi.org/10.1186/s12937-017-0298-6](https://doi.org/10.1186/s12937-017-0298-6) | No fortification |
| Johansen, N., Kondrup, J., Plum, L.M., Bak, L., Nørregaard, P., Bunch, E., Baernthsen, H., Andersen, J.R., Larsen, I.H. & Martinsen, A. (2004) Effect of nutritional support on clinical outcome in patients at nutritional risk. Clin. Nutr. 23, 539–550. | No fortification |
| [Kim, H., Kim, M., Kojima, N., Fujino, K., Hosoi, E., Kobayashi, H., Somekawa, S., Niki, Y., Yamashiro, Y., & Yoshida, H. (2016). Exercise and Nutritional Supplementation on Community-Dwelling Elderly Japanese Women With Sarcopenic Obesity: A Randomized Controlled Trial. Journal of the American Medical Directors Association, 17(11), 1011–1019. https://doi.org/10.1016/j.jamda.2016.06.016](https://doi.org/10.1016/j.jamda.2016.06.016) | No fortification |
| [Klu, Y. A. K., Phillips, R. D., & Chen, J. (2016). Development of a Drinkable, Peanut-Based Dietary Supplement and Comparison of Its Nutritional and Microbiological Qualities with Commercial Products. Journal of Food Science, 81(5), H1309-1312. https://doi.org/10.1111/1750-3841.13298](https://doi.org/10.1111/1750-3841.13298) | No fortification |
| Kouw, I.W.; Holwerda, A.M.; Trommelen, J.; Kramer, I.F.; Bastiaanse, J.; Halson, S.L.; Wodzig, W.K.; Verdijk, L.B.; van Loon, L.J. Protein Ingestion before Sleep Increases Overnight Muscle Protein Synthesis Rates in Healthy Older Men: A Randomized Controlled Trial. J. Nutr. 2017, 147, 2252–2261. | No fortification |
| Kretser, A.J., Voss, T., Kerr, W.W., Cavadini, C., Friedmann, J., 2003. Effects of two models of nutritional intervention on homebound older adults at nutritional risk. Journal of the American Dietetic Association 103, 329–336 | No fortification |
| Larsen, C.S., Toubro, S., 2007. The effect of conventional v. à la carte menu on energy and macronutrient intake among hospitalized cardiology patients. Br J Nutr 98, 351–357. | No fortification |
| Larsson, J., Unosson, M., Ek, A.-C., Nilsson, L., Thorslund, S., Bjurulf, P., 1990. Effect of dietary supplement on nutritional status and clinical outcome in 501 geriatric patients—a randomised study. Clinical Nutrition 9, 179–184. | No fortification |
| Lauque, S., 2000. Protein-energy oral supplementation in malnourished nursing-home residents. A controlled trial. Age and Ageing 29, 51–56. | No fortification |
| [Levinson, Y., Dwolatzky, T., Epstein, A., Adler, B., & Epstein, L. (2005). Is it possible to increase weight and maintain the protein status of debilitated elderly residents of nursing homes? Journals of Gerontology - Series A Biological Sciences and Medical Sciences, 60(7), 878–881. https://doi.org/10.1093/gerona/60.7.878](https://doi.org/10.1093/gerona/60.7.878) | No fortification |
| [Maltais, M. L., Perreault, K., Courchesne-Loyer, A., Lagacé, J.-C., Barsalani, R., & Dionne, I. J. (2016). Effect of resistance training and various sources of protein supplementation on body fat mass and metabolic profile in sarcopenic overweight older adult men: A pilot study. International Journal of Sport Nutrition and Exercise Metabolism, 26(1), 71–77. https://doi.org/10.1123/ijsnem.2015-0160](https://doi.org/10.1123/ijsnem.2015-0160) | No fortification |
| [Martínez-Arnau, F. M., Fonfría-Vivas, R., Buigues, C., Castillo, Y., Molina, P., Hoogland, A. J., van Doesburg, F., Pruimboom, L., Fernández-Garrido, J., & Cauli, O. (2020). Effects of leucine administration in sarcopenia: A randomized and placebo-controlled clinical trial. Nutrients, 12(4). https://doi.org/10.3390/nu12040932](https://doi.org/10.3390/nu12040932) | No fortification |
| [Massoulard, A., Bonnabau, H., Gindre-Poulvelarie, L., Baptistev, A., Preux, P. M., Villemonteix, C., Javerliat, V., Fraysse, J. L., & Desport, J. C. (2011). Analysis of the food consumption of 87 elderly nursing home residents, depending on food texture. The Journal of Nutrition, Health & Aging, 15(3), 192–195. https://doi.org/10.1007/s12603-010-0271-y](https://doi.org/10.1007/s12603-010-0271-y) | No fortification |
| [McAlpine, S., Harper, J., McMurd, M., Bolton-Smith, C., & Hetherington, M. (2003). Nutritional supplementation in older adults: Pleasantness, preference and selection of sip-feeds. In British Journal of Health Psychology (Vol. 8, Issue 1, pp. 57–66). BRITISH PSYCHOLOGICAL SOC. https://doi.org/10.1348/135910703762879200](https://doi.org/10.1348/135910703762879200) | No fortification |
| McEvoy, A. W., & James, O. F. W. (1982). The effect of a dietary supplement (build-up) on nutritional status in hospitalized elderly patients. Human Nutrition: Applied Nutrition, 36(5), 374–376. | No fortification |
| [Nilsson, M. I., Mikhail, A., Lan, L., Carlo, A. D., Hamilton, B., Barnard, K., Hettinga, B. P., Hatcher, E., Tarnopolsky, M. G., Nederveen, J. P., Bujak, A. L., May, L., & Tarnopolsky, M. A. (2020). A five-ingredient nutritional supplement and home-based resistance exercise improve lean mass and strength in free-living elderly. Nutrients, 12(8), 1–28. https://doi.org/10.3390/nu12082391](https://doi.org/10.3390/nu12082391) | No fortification |
| Norton C, Toomey C, McCormack WG, Francis P, Saunders J, Kerin E, Jakeman P. Protein supplementation at breakfast and lunch for 24 weeks beyond habitual intakes increases whole-body lean tissue mass in healthy older adults. J Nutr 2016;146:65–9. | No fortification |
| [Oikawa, S. Y., McGlory, C., D’Souza, L. K., Morgan, A. K., Saddler, N. I., Baker, S. K., Parise, G., & Phillips, S. M. (2018). A randomized controlled trial of the impact of protein supplementation on leg lean mass and integrated muscle protein synthesis during inactivity and energy restriction in older persons. American Journal of Clinical Nutrition, 108(5), 1060–1068. https://doi.org/10.1093/ajcn/nqy193](https://doi.org/10.1093/ajcn/nqy193) | No fortification |
| [Osuka, Y., Fujita, S., Kitano, N., Kosaki, K., Seol, J., Sawano, Y., Shi, H., Fujii, Y., Maeda, S., Okura, T., Kobayashi, H., & Tanaka, K. (2017). Effects of Aerobic and Resistance Training Combined with Fortified Milk on Muscle Mass, Muscle Strength, and Physical Performance in Older Adults: A Randomized Controlled Trial. The Journal of Nutrition, Health & Aging, 21(10), 1349–1357. https://doi.org/10.1007/s12603-016-0864-1](https://doi.org/10.1007/s12603-016-0864-1) | No fortification |
| Ottestad I, Lovstad AT, Gjevestad GO, Hamarsland H, Saltyte Benth J, Andersen LF, Bye A, Biong AS, Retterstol K, Iversen PO, et al. Intake of a protein-enriched milk and effects on muscle mass and strength. A 12- week randomized placebo controlled trial among community-dwelling older adults. J Nutr Health Aging 2017;21:1160–9. | No fortification |
| Payette H, Boutier V, Coulombe C. Efficacy of nutritional intervention in the free-living frail elderly. American Journal of Clinical Nutrition 2002;75(2):340S. | No fortification |
| [Pouyssegur, V., Brocker, P., Schneider, S. M., Philip, J. L., Barat, P., Reichert, E., Breugnon, F., Brunet, D., Civalleri, B., Solere, J. P., Bensussan, L., & Lupi-Pegurier, L. (2015). An innovative solid oral nutritional supplement to fight weight loss and anorexia: Open, randomised controlled trial of efficacy in institutionalised, malnourished older adults. Age and Ageing, 44(2), 245–251. https://doi.org/10.1093/ageing/afu150](https://doi.org/10.1093/ageing/afu150) | No fortification |
| [Reidy, P. T., McKenzie, A. I., Brunker, P., Nelson, D. S., Barrows, K. M., Supiano, M., Lastayo, P. C., & Drummond, M. J. (2017). Neuromuscular Electrical Stimulation Combined with Protein Ingestion Preserves Thigh Muscle Mass but Not Muscle Function in Healthy Older Adults during 5 Days of Bed Rest. Rejuvenation Research, 20(6), 449–461. https://doi.org/10.1089/rej.2017.1942](https://doi.org/10.1089/rej.2017.1942) | No fortification |
| [Reinders, I, Visser, M., & Wijnhoven, H. A. H. (2020). Two dietary advice strategies to increase protein intake among community-welling older adults: A feasibility study. In Clinical Nutrition ESPEN (Vol. 37, pp. 157–167). ELSEVIER. https://doi.org/10.1016/j.clnesp.2020.02.020](https://doi.org/10.1016/j.clnesp.2020.02.020) | No fortification |
| [Reinders, Ilse, Wijnhoven, H. A. H., Jyväkorpi, S. K., Suominen, M. H., Niskanen, R., Bosmans, J. E., Brouwer, I. A., Fluitman, K. S., Klein, M. C. A., Kuijper, L. D., van der Lubbe, L. M., Olthof, M. R., Pitkälä, K. H., Vijlbrief, R., & Visser, M. (2020). Effectiveness and cost-effectiveness of personalised dietary advice aiming at increasing protein intake on physical functioning in community-dwelling older adults with lower habitual protein intake: Rationale and design of the PROMISS randomised controlled trial. BMJ Open, 10(11), e040637. https://doi.org/10.1136/bmjopen-2020-040637](https://doi.org/10.1136/bmjopen-2020-040637) | No fortification |
| [Rondanelli, M., Cereda, E., Klersy, C., Faliva, M. A., Peroni, G., Nichetti, M., Gasparri, C., Iannello, G., Spadaccini, D., Infantino, V., Caccialanza, R., & Perna, S. (2020). Improving rehabilitation in sarcopenia: A randomized-controlled trial utilizing a muscle-targeted food for special medical purposes. Journal of Cachexia, Sarcopenia and Muscle, 11(6), 1535–1547. https://doi.org/10.1002/jcsm.12532](https://doi.org/10.1002/jcsm.12532) | No fortification |
| Rosendahl, E.; Lindelof, N.; Littbrand, H.; Yifter-Lindgren, E.; Lundin-Olsson, L.; Haglin, L.; Gustafson, Y.; Nyberg, L. High-intensity functional exercise program and protein-enriched energy supplement for older persons dependent in activities of daily living: A randomised controlled trial. Aust. J. Physiother. 2006, 52, 105–113. | No fortification |
| [Salas-Salvadó, J., Torres, M., Planas, M., Altimir, S., Pagan, C., Gonzalez, M. E., Johnston, S., Puiggros, C., Bonada, A., & García-Lorda, P. (2005). Effect of oral administration of a whole formula diet on nutritional and cognitive status in patients with Alzheimer’s disease. Clinical Nutrition (Edinburgh, Scotland), 24(3), 390–397. https://doi.org/10.1016/j.clnu.2004.12.006](https://doi.org/10.1016/j.clnu.2004.12.006) | No fortification |
| [Santos, T. D., Bastos de Freitas, B. C., Moreira, J. B., Zanfonato, K., & Vieira Costa, J. A. (2016). Development of powdered food with the addition of Spirulina for food supplementation of the elderly population. In INNOVATIVE FOOD SCIENCE & EMERGING TECHNOLOGIES (Vol. 37, Issue B, SI, pp. 216–220). ELSEVIER SCI LTD. https://doi.org/10.1016/j.ifset.2016.07.016](https://doi.org/10.1016/j.ifset.2016.07.016) | No fortification |
| Schrader, S. L., Schrank, M., Blue, R., Zawada Jr., E. T., Trujillo, A. L., & Alvai, F. K. (1995). MealMate: Improving the nutritional status of elders using a milk-based nutritional supplement. South Dakota Journal of Medicine, 48(9), 301–311. | No fortification |
| [Schürch, M.-A., Rizzoli, R., Slosman, D., Vadas, L., Vergnaud, P., & Bonjour, J.-P. (1998). Protein supplements increase serum insulin-like growth factor-I levels and attenuate proximal femur bone loss in patients with recent hip fracture. A randomized, double-blind, placebo-controlled trial. Annals of Internal Medicine, 128(10), 801–809. https://doi.org/10.7326/0003-4819-128-10-199805150-00002](https://doi.org/10.7326/0003-4819-128-10-199805150-00002) | No fortification |
| [Shahar, S., Kamaruddin, N. S., Badrasawi, M., Mohamed Sakian, N. I., Manaf, Z. A., Yassin, Z., & Joseph, L. (2013). Effectiveness of exercise and protein supplementation intervention on body composition, functional fitness, and oxidative stress among elderly Malays with sarcopenia. Clinical Interventions in Aging, 8, 1365–1375. https://doi.org/10.2147/CIA.S46826](https://doi.org/10.2147/CIA.S46826) | No fortification |
| [Song, X., Perez-Cueto, F. J. A., & Bredie, W. L. P. (2018). Sensory-Driven Development of Protein-Enriched Rye Bread and Cream Cheese for the Nutritional Demands of Older Adults. In Nutrients (Vol. 10, Issue 8). MDPI. https://doi.org/10.3390/nu10081006](https://doi.org/10.3390/nu10081006) | No fortification |
| [Song, X., Perez-Cueto, F. J. A., Laugesen, S. M. B., van der Zanden, L. D. T., & Giacalone, D. (2019). Older consumers’ attitudes towards food carriers for protein-enrichment. In APPETITE (Vol. 135, pp. 10–19). ACADEMIC PRESS LTD- ELSEVIER SCIENCE LTD. https://doi.org/10.1016/j.appet.2018.12.033](https://doi.org/10.1016/j.appet.2018.12.033) | No fortification |
| [Sugihara, Jr., P., Ribeiro, A. S., Nabuco, H. C. G., Fernandes, R. R., Tomeleri, C. M., Cunha, P. M., Venturini, D., Barbosa, D. S., Schoenfeld, B. J., & Cyrino, E. S. (2018). Effects of whey protein supplementation associated with resistance training on muscular strength, hypertrophy, and muscle quality in preconditioned older women. International Journal of Sport Nutrition and Exercise Metabolism, 28(5), 528–535. https://doi.org/10.1123/ijsnem.2017-0253](https://doi.org/10.1123/ijsnem.2017-0253) | No fortification |
| Suski, N. S., & Nielsen, C. C. (1989). Factors affecting food intake of women with Alzheimer’s type dementia in long-term care. Journal of the American Dietetic Association, 89(12), 1770–1773. | No fortification |
| Symons, T.; Sheeld-Moore, M.; Mamerow, M.; Wolfe, R.; Paddon-Jones, D. The anabolic response to resistance exercise and a protein rich meal is not diminished by age. J. Nutr. Health Aging 2011, 15, 376–381. | No fortification |
| TenHaaf DSM, Eijsvogels TMH,Bongers C,Horstman AMH,Timmers S, de Groot L, Hopman MTE. Protein supplementation improves lean body mass in physically active older adults: a randomized placebocontrolled trial. J Cachexia Sarcopenia Muscle 2019;10:298–310. | No fortification |
| [Tieland, M., van de Rest, O., Dirks, M. L., van der Zwaluw, N., Mensink, M., van Loon, L. J. C., & de Groot, L. C. P. G. M. (2012). Protein Supplementation Improves Physical Performance in Frail Elderly People: A Randomized, Double-Blind, Placebo-Controlled Trial. Journal of the American Medical Directors Association, 13(8), 720–726. https://doi.org/10.1016/j.jamda.2012.07.005](https://doi.org/10.1016/j.jamda.2012.07.005) | No fortification |
| [Torres, S. J., Robinson, S., Orellana, L., O’Connell, S. L., Grimes, C. A., Mundell, N. L., Dunstan, D. W., Nowson, C. A., & Daly, R. M. (2017). Effects of progressive resistance training combined with a protein-enriched lean red meat diet on health-related quality of life in elderly women: Secondary analysis of a 4-month cluster randomised controlled trial. The British Journal of Nutrition, 117(11), 1550–1559. https://doi.org/10.1017/S0007114517001507](https://doi.org/10.1017/S0007114517001507) | No fortification |
| [Trabal, J., Hervas, S., Forga, M., Leyes, P., & Farran-Codina, A. (2014). Usefulness of dietary enrichment on energy and protein intake in elderly patients at risk of malnutrition discharged to home. In Nutricion Hospitalaria (Vol. 29, Issue 2, pp. 382–387). AULA MEDICA EDICIONES. https://doi.org/10.3305/nh.2014.29.2.7018](https://doi.org/10.3305/nh.2014.29.2.7018) | No fortification |
| Trabal, J.; Forga, M.; Leyes, P.; Torres, F.; Rubio, J.; Prieto, E.; Farran-Codina, A. Effects of free leucine supplementation and resistance training on muscle strength and functional status in older adults: A randomized controlled trial. Clin. Interv. Aging 2015, 10, 713–723. | No fortification |
| [Tsikritzi, R., Moynihan, P. J., Gosney, M. A., Allen, V. J., & Methven, L. (2014). The effect of macro- and micro-nutrient fortification of biscuits on their sensory properties and on hedonic liking of older people. Journal of the Science of Food and Agriculture, 94(10), 2040–2048. https://doi.org/10.1002/jsfa.6522](https://doi.org/10.1002/jsfa.6522) | No fortification |
| [Tylner, S., Cederholm, T., & Faxén-Irving, G. (2016). Effects on Weight, Blood Lipids, Serum Fatty Acid Profile and Coagulation by an Energy-Dense Formula to Older Care Residents: A Randomized Controlled Crossover Trial. Journal of the American Medical Directors Association, 17(3), 275.e5-11. https://doi.org/10.1016/j.jamda.2015.12.005](https://doi.org/10.1016/j.jamda.2015.12.005) | No fortification |
| Valenzuela RE, Ponce JA, Morales-Figueroa GG, Muro KA, Carreon VR, Aleman-Mateo H. Insufficient amounts and inadequate distribution of dietary protein intake in apparently healthy older adults in a developing country: implications for dietary strategies to prevent sarcopenia. Clin Interv Aging 2013;8:1143–8. | No fortification |
| [van der Zanden, Lotte D. T., van Kleef, E., de Wijk, R. A., & van Trijp, H. C. M. (2014). Knowledge, perceptions and preferences of elderly regarding protein-enriched functional food. In Appetite (Vol. 80, pp. 16–22). ACADEMIC PRESS LTD- ELSEVIER SCIENCE LTD. https://doi.org/10.1016/j.appet.2014.04.025](https://doi.org/10.1016/j.appet.2014.04.025) | No fortification |
| [Van Wymelbeke, Virginie, Brondel, L., Bon, F., Martin-Pfitzenmeyer, I., & Manckoundia, P. (2016). An innovative brioche enriched in protein and energy improves the nutritional status of malnourished nursing home residents compared to oral nutritional supplement and usual breakfast: FARINE+ project. Clinical Nutrition ESPEN, 15, 93–100. https://doi.org/10.1016/j.clnesp.2016.06.012](https://doi.org/10.1016/j.clnesp.2016.06.012) | No fortification |
| [Vasse, E., Beelen, J., de Roos, N. M., Janssen, N., & de Groot, L. C. P. G. M. (2018). Protein intake in hospitalized older people with and without increased risk of malnutrition. In European Journal of Clinical Nutrition (Vol. 72, Issue 6, pp. 917–919). NATURE PUBLISHING GROUP. https://doi.org/10.1038/s41430-018-0171-5](https://doi.org/10.1038/s41430-018-0171-5) | No fortification |
| [Verdijk, L. B., Jonkers, R. A. M., Gleeson, B. G., Beelen, M., Meijer, K., Savelberg, H. H. C. M., Wodzig, K. W. H. W., Dendale, P., & Van Loon, L. J. C. (2009). Protein supplementation before and after exercise does not further augment skeletal muscle hypertrophy after resistance training in elderly men. American Journal of Clinical Nutrition, 89(2), 608–616. https://doi.org/10.3945/ajcn.2008.26626](https://doi.org/10.3945/ajcn.2008.26626) | No fortification |
| [Verhoeven, S., Vanschoonbeek, K., Verdijk, L. B., Koopman, R., Wodzig, W. K. W. H., Dendale, P., & Van Loon, L. J. C. (2009). Long-term leucine supplementation does not increase muscle mass or strength in healthy elderly men. American Journal of Clinical Nutrition, 89(5), 1468–1475. https://doi.org/10.3945/ajcn.2008.26668](https://doi.org/10.3945/ajcn.2008.26668) | No fortification |
| Verlaan, S.; Maier, A.B.; Bauer, J.M.; Bautmans, I.; Brandt, K.; Donini, L.M.; Maggio, M.; McMurdo, M.E.T.; Mets, T.; Seal, C.; et al. Sufficient levels of 25-hydroxyvitamin D and protein intake required to increase muscle mass in sarcopenic older adults—The PROVIDE study. Clin. Nutr. 2018, 37, 551–557. | No fortification |
| Villanueva MG, He J, Schroeder ET. Periodized resistance training with and without supplementation improve body composition and performance in older men. Eur J Appl Physiol 2014;114: 891–905. | No fortification |
| [Wouters-Wesseling, W., Slump, E., Kleijer, C. N., de Groot, L. C. P. G. M., & van Staveren, W. A. (2006). Early nutritional supplementation immediately after diagnosis of infectious disease improves body weight in psychogeriatric nursing home residents. Aging Clinical and Experimental Research, 18(1), 70–74. https://doi.org/10.1007/BF03324643](https://doi.org/10.1007/BF03324643) | No fortification |
| [Young, K.W.H., Greenwood, C.E., Van Reekum, R., Binns, M.A., 2004. Providing Nutrition Supplements to Institutionalized Seniors with Probable Alzheimer’s Disease Is Least Beneficial to Those with Low Body Weight Status: NUTRITION SUPPLEMENTS AND SENIORS WITH AD. Journal of the American Geriatrics Society 52, 1305–1312. https://doi.org/10.1111/j.1532-5415.2004.52360.x](https://doi.org/10.1111/j.1532-5415.2004.52360.x) | No fortification |
| [Zdzieblik, D., Oesser, S., Baumstark, M. W., Gollhofer, A., & König, D. (2015). Collagen peptide supplementation in combination with resistance training improves body composition and increases muscle strength in elderly sarcopenic men: A randomised controlled trial. British Journal of Nutrition, 114(8), 1237–1245. https://doi.org/10.1017/S0007114515002810](https://doi.org/10.1017/S0007114515002810) | No fortification |
| [Zhou, X., Xing, B., He, G., Lyu, X., & Zeng, Y. (2018). The Effects of Electrical Acupuncture and Essential Amino Acid Supplementation on Sarcopenic Obesity in Male Older Adults: A Randomized Control Study. Obesity Facts, 11(4), 327–334. https://doi.org/10.1159/000491797](https://doi.org/10.1159/000491797) | No fortification |
| Gray-Donald, K., Payette, H., & Boutier, V. (1995). Randomized clinical trial of nutritional supplementation shows little effect on functional status among free-living frail elderly. Journal of Nutrition, 125(12), 2965–2971. | No fortification |
| [Gray-Donald, K., Payette, H., Boutier, V., & Page, S. (1994). Evaluation of the dietary intake of homebound elderly and the feasibility of dietary supplementation. Journal of the American College of Nutrition, 13(3), 277–284. https://doi.org/10.1080/07315724.1994.10718409](https://doi.org/10.1080/07315724.1994.10718409) | No fortification |
| Hankey, C.R., Cullen, A., Wynne, H., Death, J., & Kenny, R. A. (1993). Non-starch polysaccharide/dietary fibre supplementation using small meals in long-stay frail elderly patients. European Journal of Clinical Nutrition, 47(7), 521–523. | No fortification |
| [Hegerová, P., Dědková, Z., & Sobotka, L. (2015). Early nutritional support and physiotherapy improved long-term self-sufficiency in acutely ill older patients. Nutrition, 31(1), 166–170. https://doi.org/10.1016/j.nut.2014.07.010](https://doi.org/10.1016/j.nut.2014.07.010) | No fortification |
| [Holwerda, A. M., Overkamp, M., Paulussen, K. J. M., Smeets, J. S. J., Van Kranenburg, J., Backx, E. M. P., Gijsen, A. P., Goessens, J. P. B., Verdijk, L. B., & Van Loon, L. J. C. (2018). Protein Supplementation after Exercise and before Sleep Does Not Further Augment Muscle Mass and Strength Gains during Resistance Exercise Training in Active Older Men. Journal of Nutrition, 148(11), 1723–1732. https://doi.org/10.1093/jn/nxy169](https://doi.org/10.1093/jn/nxy169) | No fortification |
| [Kemmler, W., Teschler, M., Weissenfels, A., Bebenek, M., von Stengel, S., Kohl, M., Freiberger, E., Goisser, S., Jakob, F., Sieber, C., & Engelke, K. (2016). Whole-body electromyostimulation to fight sarcopenic obesity in community-dwelling older women at risk. Resultsof the randomized controlled FORMOsA-sarcopenic obesity study. Osteoporosis International, 27(11), 3261–3270. https://doi.org/10.1007/s00198-016-3662-z](https://doi.org/10.1007/s00198-016-3662-z) | No fortification |
| [Kuosma, K., Hjerrild, J., Pedersen, P. U., & Hundrup, Y. A. (2008). Assessment of the nutritional status among residents in a Danish nursing home—Health effects of a formulated food and meal policy. Journal of Clinical Nursing, 17(17), 2288–2293. https://doi.org/10.1111/j.1365-2702.2007.02203.x](https://doi.org/10.1111/j.1365-2702.2007.02203.x) | No fortification |
| [Nabuco, H. C. G., Tomeleri, C. M., Sugihara Junior, P., Fernandes, R. R., Cavalcante, E. F., Antunes, M., Ribeiro, A. S., Teixeira, D. C., Silva, A. M., Sardinha, L. B., & Cyrino, E. S. (2018). Effects of whey protein supplementation pre- or post-resistance training on muscle mass, muscular strength, and functional capacity in pre-conditioned olderwomen: A randomized clinical trial. Nutrients, 10(5). https://doi.org/10.3390/nu10050563](https://doi.org/10.3390/nu10050563) | No fortification |
| [Wyers, C. E., Reijven, P. L. M., Breedveld-Peters, J. J. L., Denissen, K. F. M., Schotanus, M. G. M., van Dongen, M. C. J. M., Eussen, S. J. P. M., Heyligers, I. C., van den Brandt, P. A., Willems, P. C., van Helden, S., & Dagnelie, P. C. (2018). Efficacy of Nutritional Intervention in Elderly After Hip Fracture: A Multicenter Randomized Controlled Trial. The Journals of Gerontology. Series A, Biological Sciences and Medical Sciences, 73(10), 1429–1437. https://doi.org/10.1093/gerona/gly030](https://doi.org/10.1093/gerona/gly030) | No fortification |
| [Aoyama, L., Weintraub, N., & Reuben, D. B. (2005). Is weight loss in the nursing home a reversible problem? Journal of the American Medical Directors Association, 6(4), 250–256. https://doi.org/10.1016/j.jamda.2005.04.011](https://doi.org/10.1016/j.jamda.2005.04.011) | No original research |
| Avenell, A., & Handoll, H. H. (2004). Nutritional supplementation for hip fracture aftercare in the elderly. Cochrane Database of Systematic Reviews (Online), 1, CD001880. | No original research |
| [Beaudart, C., Rabenda, V., Simmons, M., Geerinck, A., Araujo de Carvalho, I., Reginster, J.-Y., Amuthavalli Thiyagarajan, J., & Bruyère, O. (2018). Effects of Protein, Essential Amino Acids, B-Hydroxy B-Methylbutyrate, Creatine, Dehydroepiandrosterone and Fatty Acid Supplementation on Muscle Mass, Muscle Strength and Physical Performance in Older People Aged 60 Years and Over. A Systematic Review of the Literature. Journal of Nutrition, Health and Aging, 22(1), 117–130. https://doi.org/10.1007/s12603-017-0934-z](https://doi.org/10.1007/s12603-017-0934-z) | No original research |
| [Blaikley, C. (2015). Use of oral nutrition supplements in the diet of malnourished older people. British Journal of Community Nursing, 20(11), 526, 528. https://doi.org/10.12968/bjcn.2015.20.11.526](https://doi.org/10.12968/bjcn.2015.20.11.526) | No original research |
| [Burgess, L. C., Phillips, S. M., & Wainwright, T. W. (2018). What is the role of nutritional supplements in support of total hip replacement and total knee replacement surgeries? A systematic review. Nutrients, 10(7). https://doi.org/10.3390/nu10070820](https://doi.org/10.3390/nu10070820) | No original research |
| Castellanos VH, Litchford MD, Campbell WW. Modular protein supplements and their application to long term care. Nutr Clin Pract. 2006;21:485-504. | No original research |
| [Cheng, H., Kong, J., Underwood, C., Petocz, P., Hirani, V., Dawson, B., & O’Leary, F. (2018). Systematic review and meta-analysis of the effect of protein and amino acid supplements in older adults with acute or chronic conditions. British Journal of Nutrition, 119(5), 527–542. https://doi.org/10.1017/S0007114517003816](https://doi.org/10.1017/S0007114517003816) | No original research |
| Churchward-Venne, T.A,; Breen, L.; Philips, S.M. Alterations in human muscle protein metabolism with aging: Protein and exercise as countermeasures to offset sarcopenia . | No original research |
| Cole D. Optimising nutrition for older people with dementia. Nurs Stand (through 2013). 2012;26(20):41–48. | No original research |
| [Cox, N. J., Ibrahim, K., Sayer, A. A., Robinson, S. M., & Roberts, H. C. (2019). Assessment and treatment of the anorexia of aging: A systematic review. Nutrients, 11(1). https://doi.org/10.3390/nu11010144](https://doi.org/10.3390/nu11010144) | No original research |
| [Donaldson, A. I. C., Smith, T. O., Alder, S., Johnstone, A. M., Roos, B. D., Aucott, L. S., Gordon, A. L., & Myint, P. K. (2019). Effect of nonmeat, high-protein supplementation on quality of life and clinical outcomes in older residents of care homes: A systematic review and meta-analysis. Nutrition Reviews, 77(2), 116–127. https://doi.org/10.1093/nutrit/nuy061](https://doi.org/10.1093/nutrit/nuy061) | No original research |
| Factors influencing the efficacy of nutritional interventions on muscle mass in older adults: A systematic review and meta-analysis | No original research |
| [Gade, J., Beck, A. M., Bitz, C., Christensen, B., Klausen, T. W., Vinther, A., & Astrup, A. (2018). Protein-enriched, milk-based supplement to counteract sarcopenia in acutely ill geriatric patients offered resistance exercise training during and after hospitalisation: Study protocol for a randomised, double-blind, multicentre trial. BMJ Open, 8(2). https://doi.org/10.1136/bmjopen-2017-019210](https://doi.org/10.1136/bmjopen-2017-019210) | No original research |
| Hankey, Catherine R., Watson, L., Lean, M., Woodward, M., & Leslie, W. (2009). IMPROVING THE DIETARY INTAKE OF FRAIL OLDER PEOPLE IN CARE HOMES USING AN ENERGY-ENRICHED FOOD APPROACH. In ANNALS OF NUTRITION AND METABOLISM (Vol. 55, Issue 1, p. 84). KARGER. | No original research |
| [Hidayat, K., Chen, G.-C., Wang, Y., Zhang, Z., Dai, X., Szeto, I. M. Y., & Qin, L.-Q. (2018). Effects of milk proteins supplementation in older adults undergoing resistance training: A meta-analysis of randomized control trials. Journal of Nutrition, Health and Aging, 22(2), 237–245. https://doi.org/10.1007/s12603-017-0899-y](https://doi.org/10.1007/s12603-017-0899-y) | No original research |
| [Holm, L., & Nordsborg, N. B. (2017). Supplementing a normal diet with protein yields a moderate improvement in the robust gains in muscle mass and strength induced by resistance training in older individuals. American Journal of Clinical Nutrition, 106(4), 971–972. https://doi.org/10.3945/ajcn.117.165860](https://doi.org/10.3945/ajcn.117.165860) | No original research |
| [Liao, C.-D., Lee, P.-H., Hsiao, D.-J., Huang, S.-W., Tsauo, J.-Y., Chen, H.-C., & Liou, T.-H. (2018). Effects of protein supplementation combined with exercise intervention on frailty indices, body composition, and physical function in frail older adults. Nutrients, 10(12). https://doi.org/10.3390/nu10121916](https://doi.org/10.3390/nu10121916) | No original research |
| [Liao, C.-D., Wu, Y.-T., Tsauo, J.-Y., Chen, P.-R., Tu, Y.-K., Chen, H.-C., & Liou, T.-H. (2020). Effects of protein supplementation combined with exercise training on muscle mass and function in older adults with lower-extremity osteoarthritis: A systematic review and meta-analysis of randomized trials. Nutrients, 12(8), 1–19. https://doi.org/10.3390/nu12082422](https://doi.org/10.3390/nu12082422) | No original research |
| [Lozano-Montoya, I., Correa-Pérez, A., Abraha, I., Soiza, R. L., Cherubini, A., O’Mahony, D., & Cruz-Jentoft, A. J. (2017). Nonpharmacological interventions to treat physical frailty and sarcopenia in older patients: A systematic overview – the SENATOR project ONTOP series. Clinical Interventions in Aging, 12, 721–740. https://doi.org/10.2147/CIA.S132496](https://doi.org/10.2147/CIA.S132496) | No original research |
| Mahoney, S., Zulli, A. & Walton, K. (2009) Patient satisfaction and energy intakes are enhanced by point of service meal provision. Nutr. Diet. 66, 212–220. | No original research |
| [Martínez-arnau, F. M., Fonfría-Vivas, R., & Cauli, O. (2019). Beneficial effects of leucine supplementation on criteria for sarcopenia: A systematic review. Nutrients, 11(10). https://doi.org/10.3390/nu11102504](https://doi.org/10.3390/nu11102504) | No original research |
| [Milne, A. C., Avenell, A., & Potter, J. (2006). Meta-analysis: Protein and energy supplementation in older people. Annals of Internal Medicine, 144(1), 37–48. https://doi.org/10.7326/0003-4819-144-1-200601030-00008](https://doi.org/10.7326/0003-4819-144-1-200601030-00008) | No original research |
| [Milne, A. C., Potter, J., Vivanti, A., & Avenell, A. (2009). Protein and energy supplementation in elderly people at risk from malnutrition. Cochrane Database of Systematic Reviews, 2. https://doi.org/10.1002/14651858.CD003288.pub3](https://doi.org/10.1002/14651858.CD003288.pub3) | No original research |
| Moon, K. T. (2012). Decreasing fall risk in older adults with nutritional intervention. American Family Physician, 86(7), 676. | No original research |
| [Newton, J., Olde Rikkert, M. G. M., & Rigaud, A.-S. (2004). Prevention of malnutrition in older people during and after hospitalisation: Results from a randomised controlled tria (multiple letters) [8]. Age and Ageing, 33(1), 87. https://doi.org/10.1093/ageing/afh025](https://doi.org/10.1093/ageing/afh025) | No original research |
| Nutritional interventions to improve muscle mass, muscle strength, and physical performance in older people: An umbrella review of systematic reviews and meta-analyses | No original research |
| [Oktaviana, J., Zanker, J., Vogrin, S., & Duque, G. (2020). The effect of protein supplements on functional frailty in older persons: A systematic review and meta-analysis. Archives of Gerontology and Geriatrics, 86. https://doi.org/10.1016/j.archger.2019.103938](https://doi.org/10.1016/j.archger.2019.103938) | No original research |
| [Porter, K. H., & Johnson, M. A. (1998). Dietary protein supplementation and recovery from femoral fracture. Nutrition Reviews, 56(11), 337–340. https://doi.org/10.1111/j.1753-4887.1998.tb01672.x](https://doi.org/10.1111/j.1753-4887.1998.tb01672.x) | No original research |
| Smith, G.I. The Effects of Dietary Omega-3s on Muscle Composition and Quality in Older Adults. Curr. Nutr. Rep. 2016, 5, 99–105. | No original research |
| Stratton RJ. Should food or supplements be used in the community for the treatment of disease-related malnutrition? Proc Nutr Soc 2005;64:325–33. | No original research |
| The efficacy of essential amino acid supplementation for augmenting dietary protein intake in older adults: Implications for skeletal muscle mass, strength and function | No original research |
| [Thomas, D. K., Quinn, M. A., Saunders, D. H., & Greig, C. A. (2016). Protein Supplementation Does Not Significantly Augment the Effects of Resistance Exercise Training in Older Adults: A Systematic Review. Journal of the American Medical Directors Association, 17(10), 959.e1-959.e9. https://doi.org/10.1016/j.jamda.2016.07.002](https://doi.org/10.1016/j.jamda.2016.07.002) | No original research |
| [Valenzuela, P. L., Mata, F., Morales, J. S., Castillo-García, A., & Lucia, A. (2019). Does beef protein supplementation improve body composition and exercise performance? A systematic review and meta-analysis of randomized controlled trials. Nutrients, 11(6). https://doi.org/10.3390/nu11061429](https://doi.org/10.3390/nu11061429) | No original research |
| [Wirth, J., Hillesheim, E., & Brennan, L. (2020). The Role of Protein Intake and its Timing on Body Composition and Muscle Function in Healthy Adults: A Systematic Review and Meta-Analysis of Randomized Controlled Trials. Journal of Nutrition, 150(6), 1443–1460. https://doi.org/10.1093/jn/nxaa049](https://doi.org/10.1093/jn/nxaa049) | No original research |
| [Baugreet, S., Kerry, J. P., Allen, P., & Hamill, R. M. (2017). Optimisation of protein-fortified beef patties targeted to the needs of older adults: A mixture design approach. Meat Science, 134, 111–118. https://doi.org/10.1016/j.meatsci.2017.07.023](https://doi.org/10.1016/j.meatsci.2017.07.023) | Wrong outcome |
| [Baugreet, S., Kerry, J. P., Botineştean, C., Allen, P., & Hamill, R. M. (2016). Development of novel fortified beef patties with added functional protein ingredients for the elderly. Meat Science, 122, 40–47. https://doi.org/10.1016/j.meatsci.2016.07.004](https://doi.org/10.1016/j.meatsci.2016.07.004) | Wrong outcome |
| [Björkman, M. P., Suominen, M. H., Kautiainen, H., Jyväkorpi, S. K., Finne-Soveri, H. U., Strandberg, T. E., Pitkälä, K. H., & Tilvis, R. S. (2020). Effect of Protein Supplementation on Physical Performance in Older People With Sarcopenia–A Randomized Controlled Trial. Journal of the American Medical Directors Association, 21(2), 226-232.e1. https://doi.org/10.1016/j.jamda.2019.09.006](https://doi.org/10.1016/j.jamda.2019.09.006) | Wrong outcome |
| [Coker, R. H., Shin, K., Scholten, K., Johannsen, M., Tsigonis, J., Kim, I.-Y., Schutzler, S. E., & Wolfe, R. R. (2019). Essential amino acid-enriched meal replacement promotes superior net protein balance in older, overweight adults. Clinical Nutrition, 38(6), 2821–2826. https://doi.org/10.1016/j.clnu.2018.12.013](https://doi.org/10.1016/j.clnu.2018.12.013) | Wrong outcome |
| [Devries, M. C., McGlory, C., Bolster, D. R., Kamil, A., Rahn, M., Harkness, L., Baker, S. K., & Phillips, S. M. (2018). Protein leucine content is a determinant of shorter-and longer-term muscle protein synthetic responses at rest and following resistance exercise in healthy older women: A randomized, controlled trial. American Journal of Clinical Nutrition, 107(2), 217–226. https://doi.org/10.1093/ajcn/nqx028](https://doi.org/10.1093/ajcn/nqx028) | Wrong outcome |
| [Giezenaar, C., Lange, K., Hausken, T., Jones, K. L., Horowitz, M., Chapman, I., & Soenen, S. (2020). Effects of age on acute appetite-related responses to whey-protein drinks, including energy intake, gastric emptying, blood glucose, and plasma gut hormone concentrations—A randomized controlled trial. Nutrients, 12(4). https://doi.org/10.3390/nu12041008](https://doi.org/10.3390/nu12041008) | Wrong outcome |
| [Hameed, R. H. (2018). The effect of vitamin d and leucine—Enriched whey protein nutritional supplement on measures of sarcopenia in older adults. Indian Journal of Public Health Research and Development, 9(12), 1074–1079. https://doi.org/10.5958/0976-5506.2018.01992.7](https://doi.org/10.5958/0976-5506.2018.01992.7) | Wrong outcome |
| Höglund E, Albinsson B, Stuhr-Olsson G, Signäs M, Karlsson C, Rothenberg E and Wendin K. Protein and Energy Enriched Muffins Designed for Nutritional Needs of Older Adults, Nutrition & Food Science International Journal 2(4). (2017). | Wrong outcome |
| [Kemmler, W., Kohl, M., Jakob, F., Engelke, K., & von Stengel, S. (2020). Effects of high intensity dynamic resistance exercise and whey protein supplements on osteosarcopenia in older men with low bone and muscle Mass. Final results of the randomized controlled FrOST study. Nutrients, 12(8), 1–18. https://doi.org/10.3390/nu12082341](https://doi.org/10.3390/nu12082341) | Wrong outcome |
| [Kerstetter, J. E., Bihuniak, J. D., Brindisi, J., Sullivan, R. R., Mangano, K. M., Larocque, S., Kotler, B. M., Simpson, C. A., Cusano, A. M., Gaffney-Stomberg, E., Kleppinger, A., Reynolds, J., Dziura, J., Kenny, A. M., & Insogna, K. L. (2015). The effect of a whey protein supplement on bone mass in older Caucasian adults. Journal of Clinical Endocrinology and Metabolism, 100(6), 2214–2222. https://doi.org/10.1210/jc.2014-3792](https://doi.org/10.1210/jc.2014-3792) | Wrong outcome |
| [Kramer, I. F., Verdijk, L. B., Hamer, H. M., Verlaan, S., Luiking, Y. C., Kouw, I. W. K., Senden, J. M., van Kranenburg, J., Gijsen, A. P., Bierau, J., Poeze, M., & van Loon, L. J. C. (2017). Both basal and post-prandial muscle protein synthesis rates, following the ingestion of a leucine-enriched whey protein supplement, are not impaired in sarcopenic older males. Clinical Nutrition (Edinburgh, Scotland), 36(5), 1440–1449. https://doi.org/10.1016/j.clnu.2016.09.023](https://doi.org/10.1016/j.clnu.2016.09.023) | Wrong outcome |
| [Kreijkamp-Kaspers, S., Kok, L., Grobbee, D. E., De Haan, E. H. F., Aleman, A., Lampe, J. W., & Van Der Schouw, Y. T. (2004). Effect of soy protein containing isoflavones on cognitive function, bone mineral density, and plasma lipilds in postmenopausal women: A randomized controlled trial. Journal of the American Medical Association, 292(1), 65–74. https://doi.org/10.1001/jama.292.1.65](https://doi.org/10.1001/jama.292.1.65) | Wrong outcome |
| [Murphy, C. H., Saddler, N. I., Devries, M. C., McGlory, C., Baker, S. K., & Phillips, S. M. (2016). Leucine supplementation enhances integrative myofibrillar protein synthesis in free-living older men consuming lower-and higher-protein diets: A parallel-group crossover study1. American Journal of Clinical Nutrition, 104(6), 1594–1606. https://doi.org/10.3945/ajcn.116.136424](https://doi.org/10.3945/ajcn.116.136424) | Wrong outcome |
| [Neelemaat, F., Bosmans, J. E., Thijs, A., Seidell, J. C., & van Bokhorst-de van der Schueren, M. A. E. (2011). Post-Discharge Nutritional Support in Malnourished Elderly Individuals Improves Functional Limitations. In JOURNAL OF THE AMERICAN MEDICAL DIRECTORS ASSOCIATION (Vol. 12, Issue 4, pp. 295–301). ELSEVIER SCIENCE INC. https://doi.org/10.1016/j.jamda.2010.12.005](https://doi.org/10.1016/j.jamda.2010.12.005) | Wrong outcome |
| [Neelemaat, F., Bosmans, J. E., Thijs, A., Seidell, J. C., & van Bokhorst-de van der Schueren, M. A. E. (2012). Oral nutritional support in malnourished elderly decreases functional limitations with no extra costs. In Clinical Nutrition (Vol. 31, Issue 2, pp. 183–190). CHURCHILL LIVINGSTONE. https://doi.org/10.1016/j.clnu.2011.10.009](https://doi.org/10.1016/j.clnu.2011.10.009) | Wrong outcome |
| [Pouysségur, V., Pouysségur, V., Castelli, C., Chkair, S., Bouvet, S., Castelli, C., Chkair, S., & Bouvet, S. (2017). Solid oral supplementation: Economic assessment. Economic impact of the introduction of a solid oral nutritional supplement adapted to malnourished older adults with poor dental health. European Geriatric Medicine, 8(3), 234–239. https://doi.org/10.1016/j.eurger.2017.04.012](https://doi.org/10.1016/j.eurger.2017.04.012) | Wrong outcome |
| Szcześniak, D., Budzeń, S., Kopeć, W. & Rymaszewska, J. Anserine and carnosine supplementation in the elderly: Effects on cognitive functioning and physical capacity. Arch. Gerontol. Geriatr. 59, 485–90 2014 | Wrong outcome |
| [van der Zanden, L.D.T., van Kleef, E., de Wijk, R. A., & van Trijp, H. C. M. (2015). Examining heterogeneity in elderly consumers’ acceptance of carriers for protein-enriched food: A segmentation study. Food Quality and Preference, 42, 130–138. https://doi.org/10.1016/j.foodqual.2015.01.016](https://doi.org/10.1016/j.foodqual.2015.01.016) | Wrong outcome |
| [Zhu, K., Meng, X., Kerr, D. A., Devine, A., Solah, V., Binns, C. W., & Prince, R. L. (2011). The effects of a two-year randomized, controlled trial of whey protein supplementation on bone structure, IGF-1, and urinary calcium excretion in older postmenopausal women. Journal of Bone and Mineral Research, 26(9), 2298–2306. https://doi.org/10.1002/jbmr.429](https://doi.org/10.1002/jbmr.429) | Wrong outcome |
| [Zhu, Kun, Kerr, D. A., Meng, X., Devine, A., Solah, V., Binns, C. W., & Prince, R. L. (2015). Two-Year Whey Protein Supplementation Did Not Enhance Muscle Mass and Physical Function in Well-Nourished Healthy Older Postmenopausal Women. In Journal of Nutrition (Vol. 145, Issue 11, pp. 2520–2526). OXFORD UNIV PRESS. https://doi.org/10.3945/jn.115.218297](https://doi.org/10.3945/jn.115.218297) | Wrong outcome |
| [Arjmandi, B. H., Lucas, E. A., Khalil, D. A., Devareddy, L., Smith, B. J., McDonald, J., Arquitt, A. B., Payton, M. E., & Mason, C. (2005). One year soy protein supplementation has positive effects on bone formation markers but not bone density in postmenopausal women. Nutrition Journal, 4. https://doi.org/10.1186/1475-2891-4-8](https://doi.org/10.1186/1475-2891-4-8) | Wrong population |
| [Bemben, M. G., Witten, M. S., Carter, J. M., Eliot, K. A., Knehans, A. W., & Bemben, D. A. (2010). The effects of supplementation with creatine and protein on muscle strength following a traditional resistance training program in middle-aged and older men. Journal of Nutrition, Health and Aging, 14(2), 155–159. https://doi.org/10.1007/s12603-009-0124-8](https://doi.org/10.1007/s12603-009-0124-8) | Wrong population |
| [Bohl, M., Bjørnshave, A., Larsen, M. K., Gregersen, S., & Hermansen, K. (2017). The effects of proteins and medium-chain fatty acids from milk on body composition, insulin sensitivity and blood pressure in abdominally obese adults. European Journal of Clinical Nutrition, 71(1), 76–82. https://doi.org/10.1038/ejcn.2016.207](https://doi.org/10.1038/ejcn.2016.207) | Wrong population |
| [Cecchi, L., Schuster, N., Flynn, D., Bechtel, R., Bellumori, M., Innocenti, M., Mulinacci, N., & Guinard, J.-X. (2019). Sensory Profiling and Consumer Acceptance of Pasta, Bread, and Granola Bar Fortified with Dried Olive Pomace (Pâté): A Byproduct from Virgin Olive Oil Production. Journal of Food Science, 84(10), 2995–3008. https://doi.org/10.1111/1750-3841.14800](https://doi.org/10.1111/1750-3841.14800) | Wrong population |
| [Chen, Y., Zhang, Q., Wang, Y., Xiao, Y., Fu, R., Bao, H., & Liu, M. (2015). Estimating the causal effect of milk powder supplementation on bone mineral density: A randomized controlled trial with both non-compliance and loss to follow-up. European Journal of Clinical Nutrition, 69(7), 824–830. https://doi.org/10.1038/ejcn.2015.3](https://doi.org/10.1038/ejcn.2015.3) | Wrong population |
| [Daly, R. M., Gianoudis, J., De Ross, B., O’Connell, S. L., Kruger, M., Schollum, L., & Gunn, C. (2020). Effects of a multinutrient-fortified milk drink combined with exercise on functional performance, muscle strength, body composition, inflammation, and oxidative stress in middle-aged women: A 4-month, double-blind, placebo-controlled, randomized trial. The American Journal of Clinical Nutrition, 112(2), 427–446. https://doi.org/10.1093/ajcn/nqaa126](https://doi.org/10.1093/ajcn/nqaa126) | Wrong population |
| [Kamo, N., Kaido, T., Uozumi, R., Ito, T., Yagi, S., Hata, K., Taura, K., & Uemoto, S. (2020). Effect of administration of β-hydroxy-β-methyl butyrate-enriched formula after liver transplantation: A pilot randomized controlled trial. Nutrition, 79–80. https://doi.org/10.1016/j.nut.2020.110871](https://doi.org/10.1016/j.nut.2020.110871) | Wrong population |
| [Kang, Y., Kim, N., Choi, Y. J., Lee, Y., Yun, J., Park, S. J., Park, H. S., Chung, Y.-S., & Park, Y. K. (2020). Leucine-enriched protein supplementation increases lean body mass in healthy Korean adults aged 50 years and older: A randomized, double-blind, placebo-controlled trial. Nutrients, 12(6), 1–16. https://doi.org/10.3390/nu12061816](https://doi.org/10.3390/nu12061816) | Wrong population |
| [Matthews, L. E. (1991). Nutrition intervention in the frail elderly—A case study. Journal of Nutrition for the Elderly, 10(3), 63–68. https://doi.org/10.1300/J052v10n03_05](https://doi.org/10.1300/J052v10n03_05) | Wrong population |
| [Ortega, J. F., Morales-Palomo, F., Fernandez-Elias, V., Hamouti, N., Bernardo, F. J., Martin-Doimeadios, R. C., Nelson, R. K., Horowitz, J. F., & Mora-Rodriguez, R. (2016). Dietary supplementation with omega-3 fatty acids and oleate enhances exercise training effects in patients with metabolic syndrome. Obesity, 24(8), 1704–1711. https://doi.org/10.1002/oby.21552](https://doi.org/10.1002/oby.21552) | Wrong population |
| Protein-enriched diet improved muscle endurance and marginally reduced intramuscular adiposity: Results from a randomized controlled trial among middle-aged and older adults. | Wrong population |
| [Sammarco, R., Marra, M., Di Guglielmo, M. L., Naccarato, M., Contaldo, F., Poggiogalle, E., Donini, L. M., & Pasanisi, F. (2017). Evaluation of Hypocaloric Diet with Protein Supplementation in Middle-Aged Sarcopenic Obese Women: A Pilot Study. Obesity Facts, 10(3), 160–167. https://doi.org/10.1159/000468153](https://doi.org/10.1159/000468153) | Wrong population |
| Saudny-Unterberger H, Martin JG, Gray-Donald K. Impact of nutritional support on functional status during acute exacerbation of chronic obstructive pulmonary disease. American Journal of Respiratory and Critical Care Medicine 1997;156:794-9. | Wrong population |
| [Smith, G. I., Commean, P. K., Reeds, D. N., Klein, S., & Mittendorfer, B. (2018). Effect of Protein Supplementation During Diet-Induced Weight Loss on Muscle Mass and Strength: A Randomized Controlled Study. Obesity, 26(5), 854–861. https://doi.org/10.1002/oby.22169](https://doi.org/10.1002/oby.22169) | Wrong population |
| [Thomas, S. D. C., Morris, H. A., & Nordin, B. E. C. (2015). Acute effect of a supplemented milk drink on bone metabolism in healthy postmenopausal women is influenced by the metabolic syndrome. Nutrition Journal, 14(1), 99. https://doi.org/10.1186/s12937-015-0092-2](https://doi.org/10.1186/s12937-015-0092-2) | Wrong population |
